# Supplementary material for: Single-cell analysis of signalling and transcriptional responses to type I interferons
Source: EMBO Rep. 2026 Mar 27;27(9):2491–513. doi: 10.1038/s44319-026-00750-3 (PMC13172048; doi:10.1038/s44319-026-00750-3)
Supplement: Supplementary file 1 — Appendix [file 44319_2026_750_MOESM1_ESM.pdf]

Appendix

Single-cell analysis of signalling and transcriptional responses to type I interferons

Rachel E. Rigby[1], Kevin Rue-Albrecht[2], Aleksandr Fedorov[1], David Sims[2] and Jan Rehwinkel[1*]

[1]MRC Translational Immune Discovery Unit, MRC Weatherall Institute of Molecular Medicine, Radcliffe Department of Medicine, University of Oxford, Oxford, UK
[2]MRC WIMM Centre for Computational Biology, MRC Weatherall Institute of Molecular Medicine, University of Oxford, Oxford, UK

*Correspondence to: jan.rehwinkel@imm.ox.ac.uk.

Table of Content

Appendix Figure	Page
S1	2-3
S2	4
S3	5-6
S4	7
S5	8-9
S6	10-11
S7	12
S8	13-14
S9	15
S10	16-17
S11	18-19
S12	20-21
S13	22
S14	23
S15	24-25
S16	26
S17	27
S18	28-29
S19	30-31
S20	32
S21	33
S22	34
S23	35
S24	36
S25	37
S26	38
S27	39-40

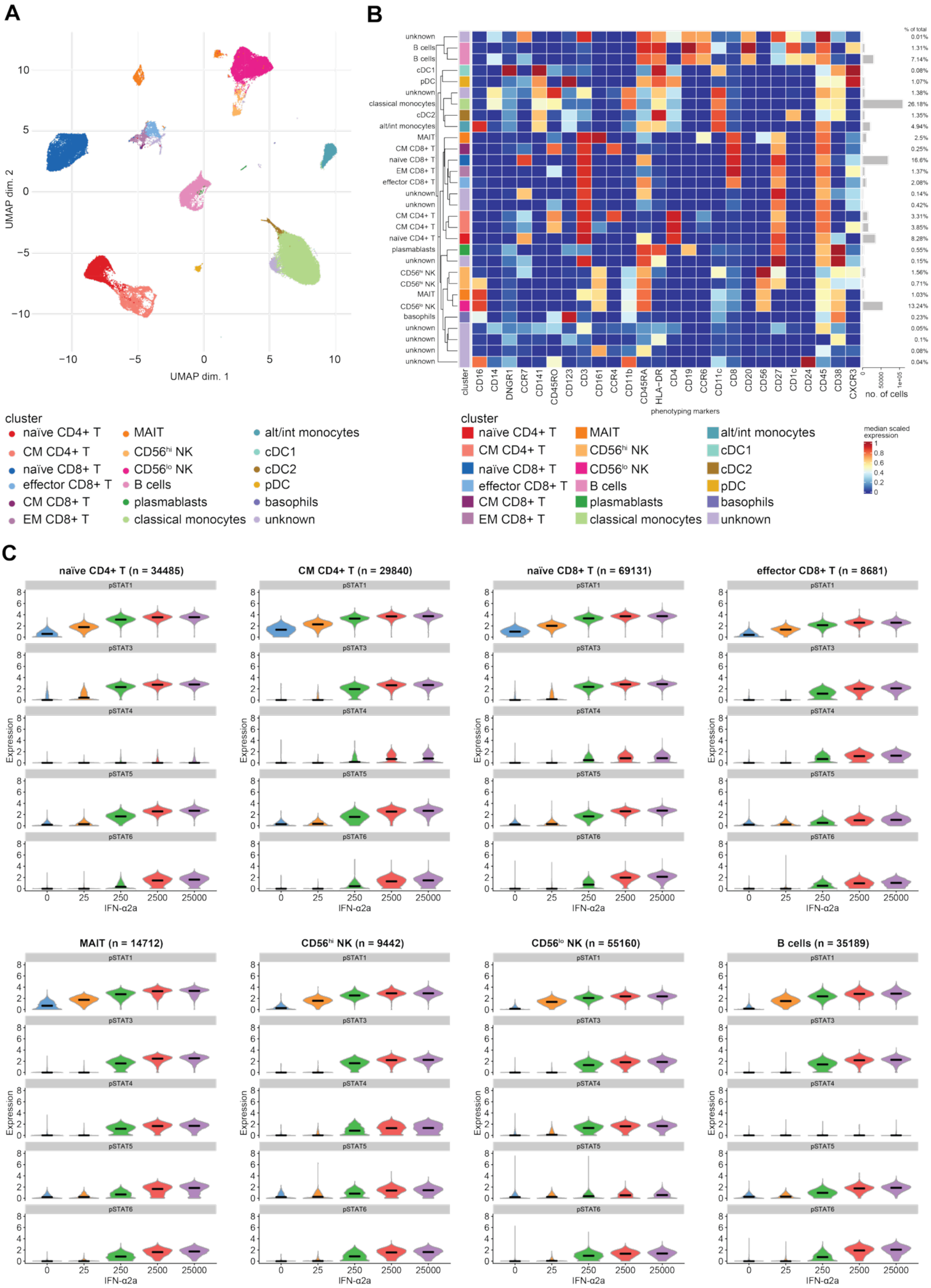
A
B
C
UMAP dim. 1
UMAP dim. 2
cluster
naïve CD4+ T
CM CD4+ T
naïve CD8+ T
effector CD8+ T
CM CD8+ T
EM CD8+ T
MAIT
CD56hi NK
CD56lo NK
B cells
plasmablasts
classical monocytes
alt/int monocytes
cDC1
cDC2
pDC
basophils
unknown
phenotyping markers
no. of cells
% of total
median scaled expression
naïve CD4+ T (n = 34485)
CM CD4+ T (n = 29840)
naïve CD8+ T (n = 69131)
effector CD8+ T (n = 8681)
MAIT (n = 14712)
CD56hi NK (n = 9442)
CD56lo NK (n = 55160)
B cells (n = 35189)
pSTAT1
pSTAT3
pSTAT4
pSTAT5
pSTAT6
Expression
IFN-α2a

C - continued

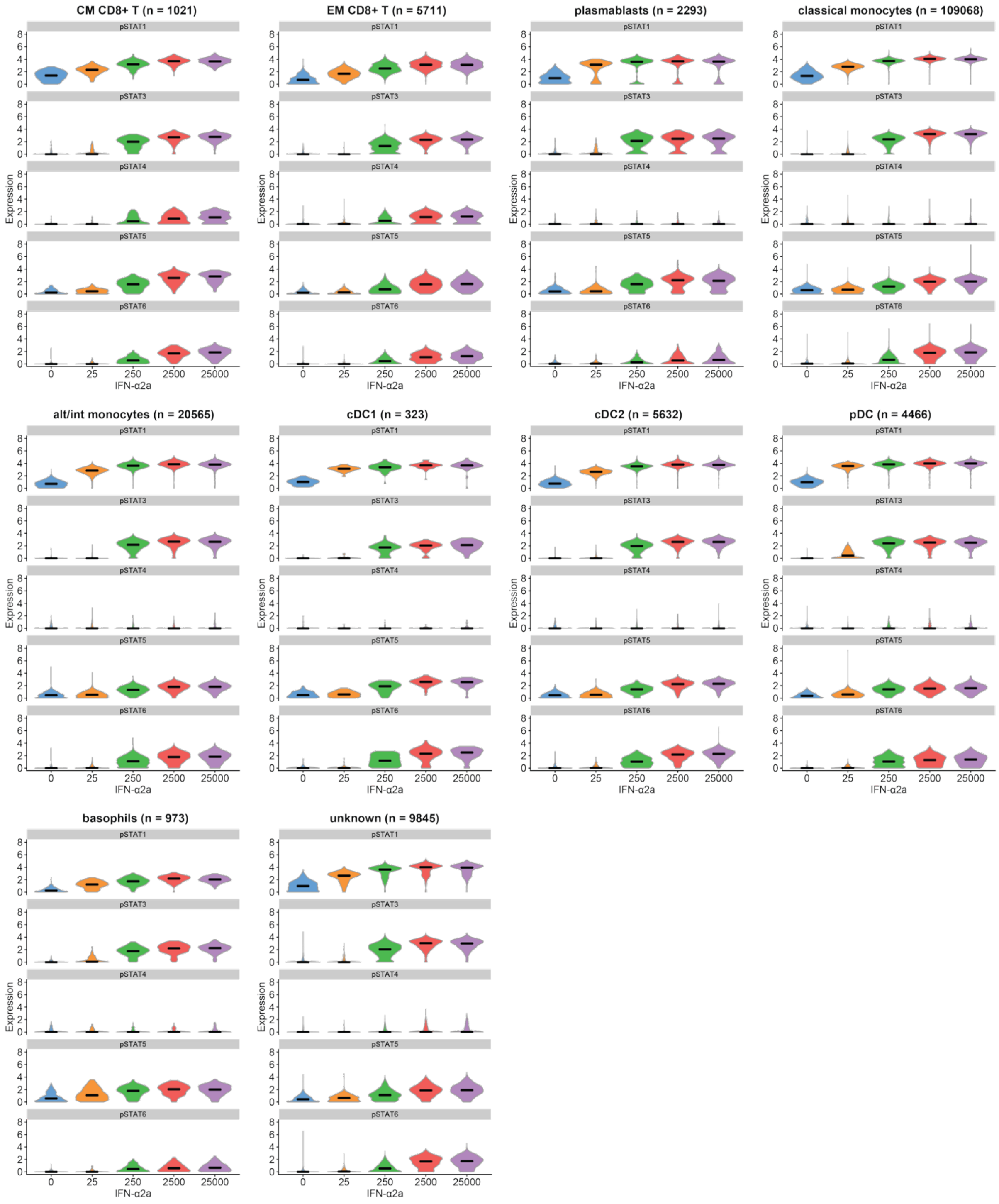

Appendix Figure S1 (Related to Figure 1). Phosphorylation of STAT proteins in response to IFN-α2a at 15 minutes

(A, B) UMAP (A) and heatmap (B) plots showing clustering of PBMCs and identification of different cell types based on expression of phenotyping markers. The percentage of cells per cluster is shown in the histogram on the right-hand side of (B). (C) Violin plots showing expression of each pSTAT in the indicated cell types in response to increasing concentrations of IFN-α2a.

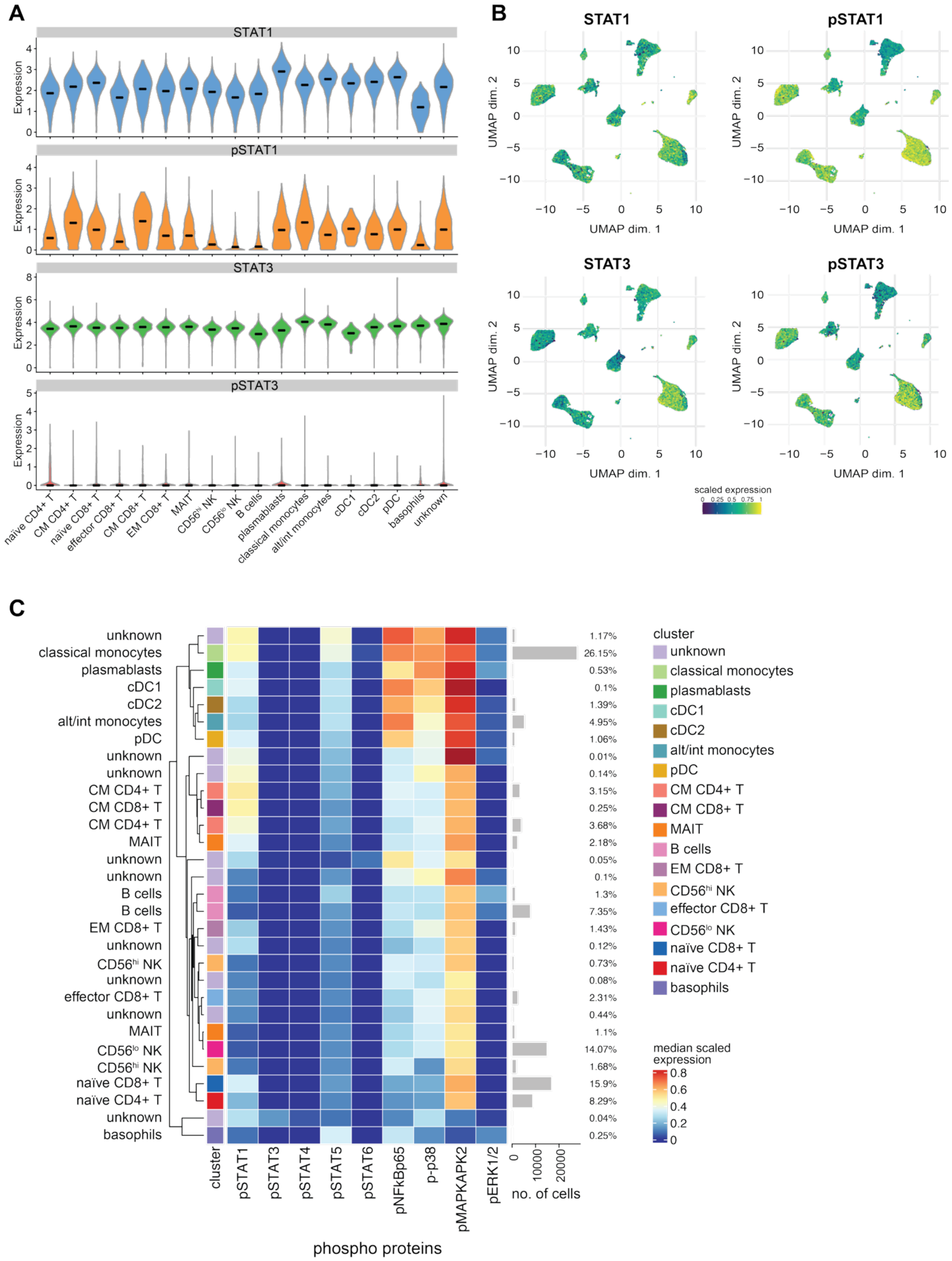

Appendix Figure S2 (Related to Figure 1). Baseline expression of STAT1, pSTAT1, STAT3 and pSTAT3 in different cell types

(A, B) Violin (A) and UMAP (B) plots showing the expression of STAT1, pSTAT1, STAT3 and pSTAT3 in unstimulated PBMCs, clustered as shown in Appendix Figure S1A. (C) Heatmap showing expression of phosphorylated proteins in unstimulated cells.

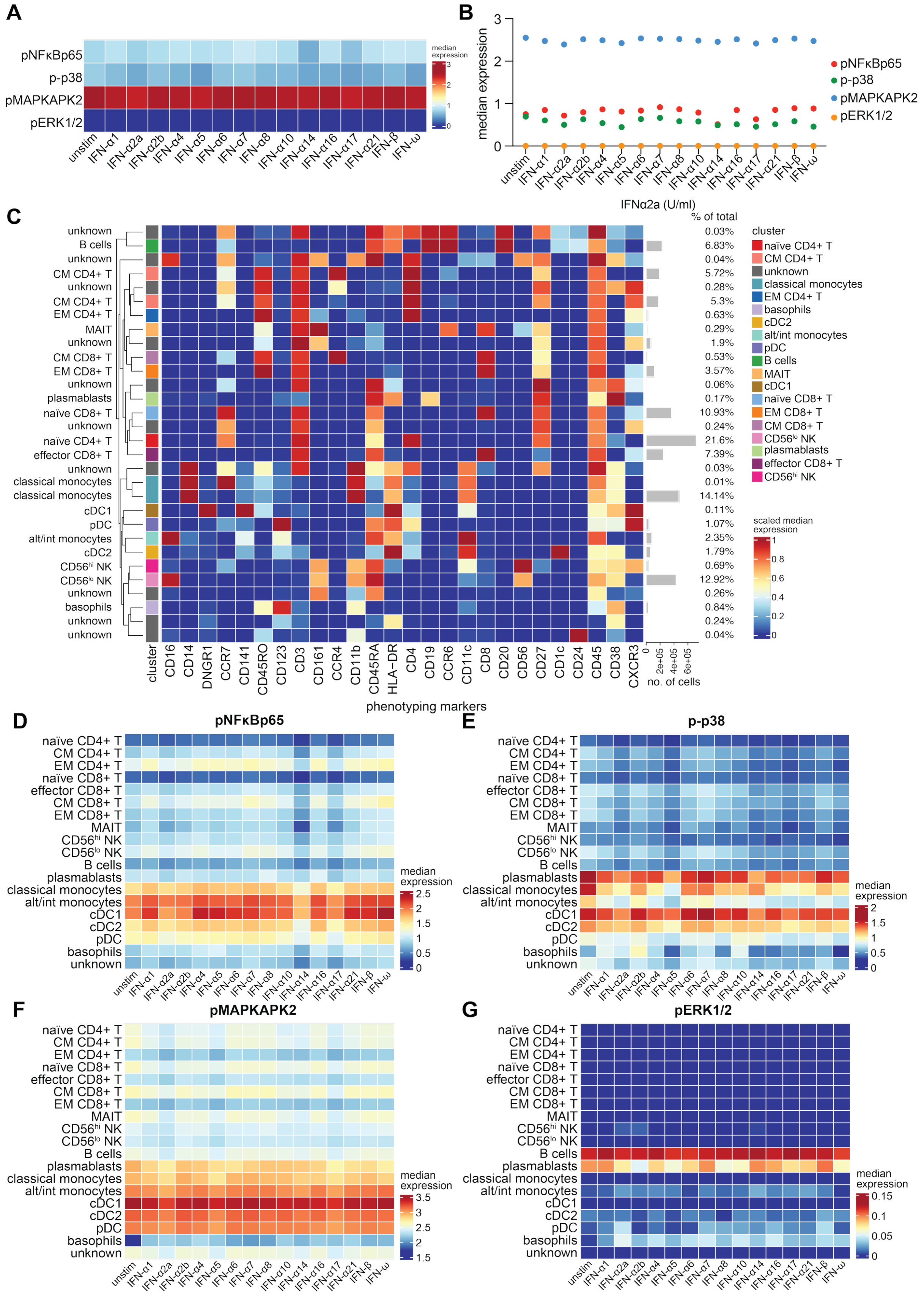
A
pNFκBp65
p-p38
pMAPKAPK2
pERK1/2
median expression
unstim
IFN-α1
IFN-α2a
IFN-α2b
IFN-α4
IFN-α5
IFN-α6
IFN-α7
IFN-α8
IFN-α10
IFN-α14
IFN-α16
IFN-α17
IFN-α21
IFN-β
IFN-ω
B
median expression
IFNα2a (U/ml)
C
% of total
cluster
naïve CD4+ T
CM CD4+ T
unknown
classical monocytes
EM CD4+ T
basophils
cDC2
alt/int monocytes
pDC
B cells
MAIT
cDC1
naïve CD8+ T
EM CD8+ T
CM CD8+ T
CD56lo NK
plasmablasts
effector CD8+ T
CD56hi NK
scaled median expression
no. of cells
phenotyping markers
CD16
CD14
DNGR1
CCR7
CD141
CD45RO
CD123
CD3
CD161
CCR4
CD11b
CD45RA
HLA-DR
CD4
CD19
CCR6
CD11c
CD8
CD20
CD56
CD27
CD1c
CD24
CD45
CD38
CXCR3
D
pNFκBp65
E
p-p38
F
pMAPKAPK2
G
pERK1/2

Appendix Figure S3 (Related to Figure 2). Phosphorylation of signalling proteins in response to type I IFN stimulation at 15 minutes

(A) Median expression of pNFκBp65, p-p38, pMAPKAPK2 and pERK1/2 in PBMCs in response to treatment with 2,500 U/ml of each type I IFN for 15 minutes. (B) Depiction of the data shown in (A) as a dot plot. (C) Heatmap showing expression of the 26 phenotyping markers used for cell type identification as shown in Figure 2C. The percentage of cells per cluster is shown in the histogram on the right-hand side. (D-G) Heatmaps showing median expression of the indicated phosphoproteins in each cell type in response to treatment with different type I IFNs.

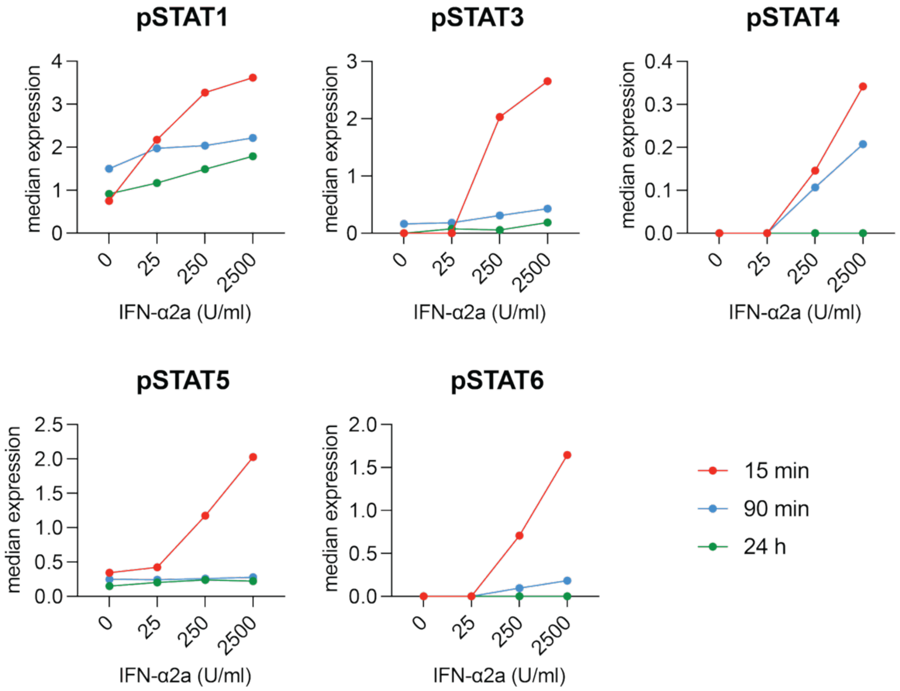

Appendix Figure S4 (Related to Figure 2). Phosphorylation of STAT proteins in response to IFN-α2a at different timepoints

Median expression of pSTATs in total PBMCs after stimulation with IFN-α2a for 15 minutes, 90 minutes or 24 hours. The data were pooled from Figures 1C, S5B and S6B without further normalisation.

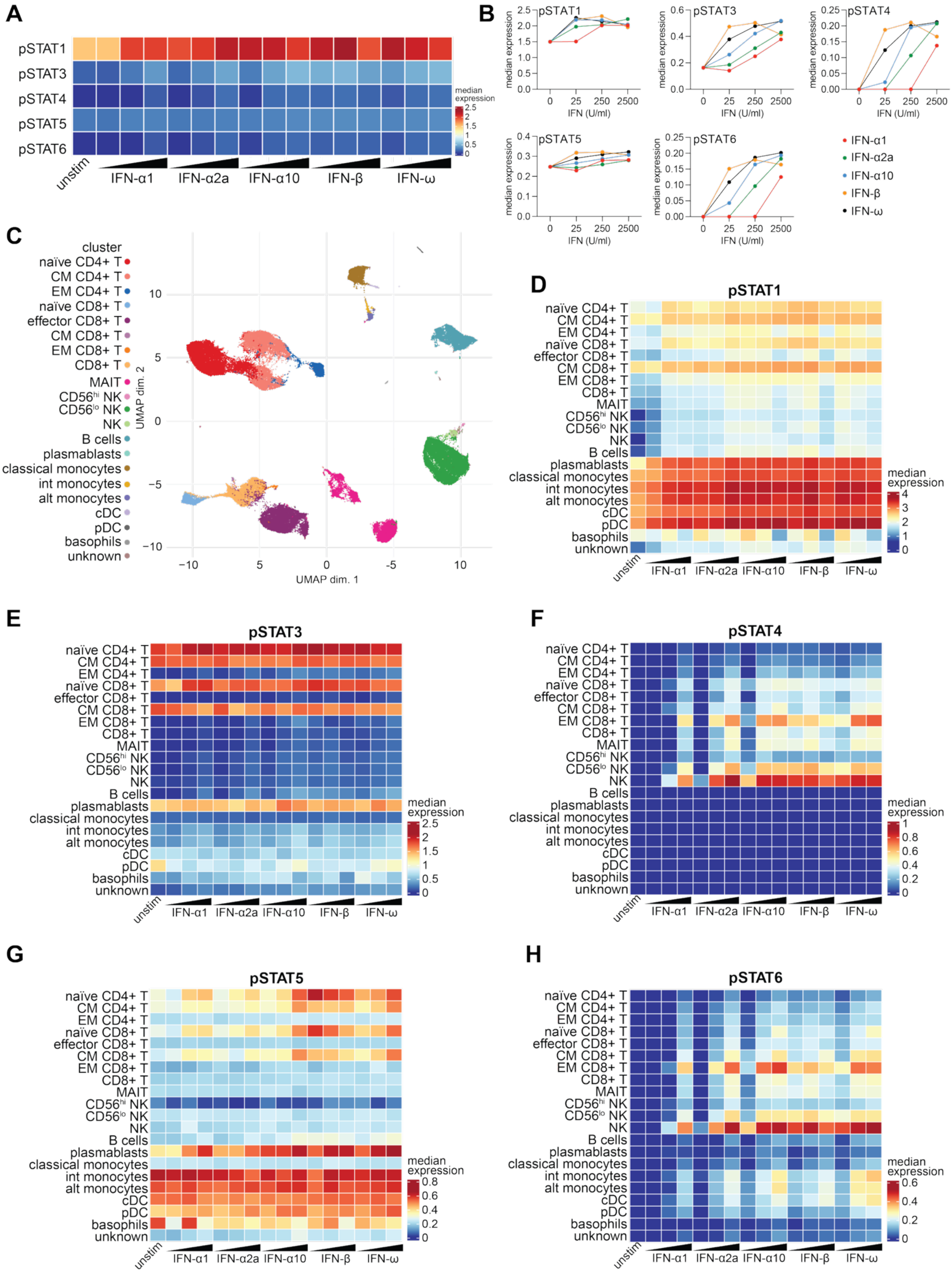
A
pSTAT1
pSTAT3
pSTAT4
pSTAT5
pSTAT6
unstim
IFN-α1
IFN-α2a
IFN-α10
IFN-β
IFN-ω
median expression
B
pSTAT1
pSTAT3
pSTAT4
pSTAT5
pSTAT6
median expression
IFN (U/ml)
IFN-α1
IFN-α2a
IFN-α10
IFN-β
IFN-ω
C
cluster
naïve CD4+ T
CM CD4+ T
EM CD4+ T
naïve CD8+ T
effector CD8+ T
CM CD8+ T
EM CD8+ T
CD8+ T
MAIT
CD56hi NK
CD56lo NK
NK
B cells
plasmablasts
classical monocytes
int monocytes
alt monocytes
cDC
pDC
basophils
unknown
UMAP dim. 1
UMAP dim. 2
D
pSTAT1
E
pSTAT3
F
pSTAT4
G
pSTAT5
H
pSTAT6

Appendix Figure S5 (Related to Figure 2). Phosphorylation of STAT proteins in response to stimulation with type I IFNs at 90 minutes

(A) Median expression of pSTATs in PBMCs in response to treatment with 25, 250 and 2,500 U/ml of the indicated type I IFNs for 90 minutes. (B) Depiction of the data shown in (A) as line plots. (C) UMAP plot showing clustering of PBMCs and identification of different cell types based on expression of the phenotyping markers. (D-H) Heatmaps showing median expression of pSTATs in each cell type in response to treatment with different type I IFNs.

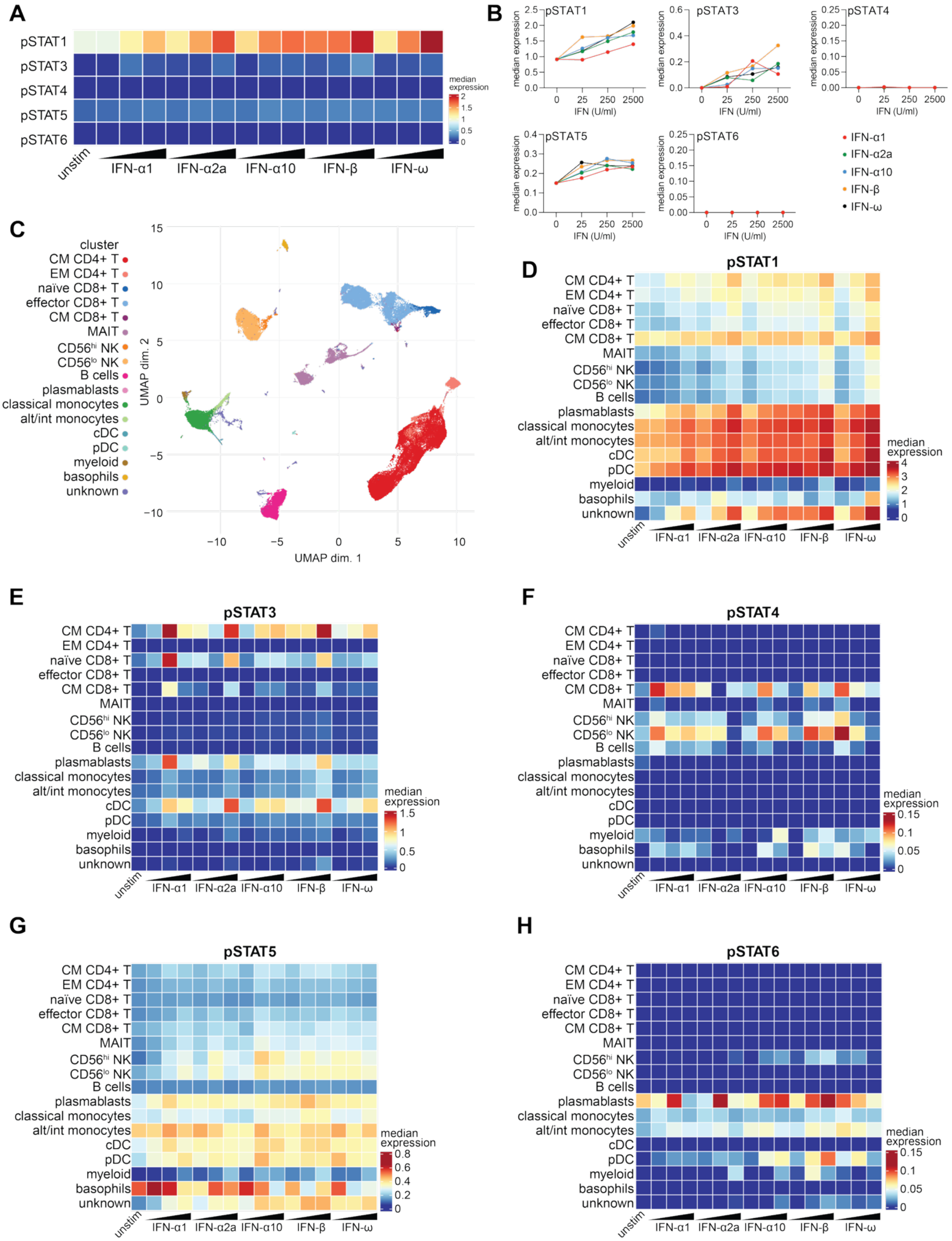

A
pSTAT1
pSTAT3
pSTAT4
pSTAT5
pSTAT6
unstim
IFN-α1
IFN-α2a
IFN-α10
IFN-β
IFN-ω
median expression
B
pSTAT1
pSTAT3
pSTAT4
pSTAT5
pSTAT6
median expression
IFN (U/ml)
IFN-α1
IFN-α2a
IFN-α10
IFN-β
IFN-ω
C
cluster
CM CD4+ T
EM CD4+ T
naïve CD8+ T
effector CD8+ T
CM CD8+ T
MAIT
CD56hi NK
CD56lo NK
B cells
plasmablasts
classical monocytes
alt/int monocytes
cDC
pDC
myeloid
basophils
unknown
UMAP dim. 1
UMAP dim. 2
D
pSTAT1
E
pSTAT3
F
pSTAT4
G
pSTAT5
H
pSTAT6

Appendix Figure S6 (Related to Figure 2). Phosphorylation of STAT proteins in response to stimulation with type I IFNs at 24 hours

(A) Median expression of pSTATs in PBMCs in response to treatment with 25, 250 and 2,500 U/ml of the indicated type I IFNs for 24 hours. (B) Depiction of the data shown in (A) as line plots. (C) UMAP plot showing clustering of PBMCs and identification of different cell types based on expression of the phenotyping markers. (D-H) Heatmaps showing median expression of pSTATs in each cell type in response to treatment with different type I IFNs.

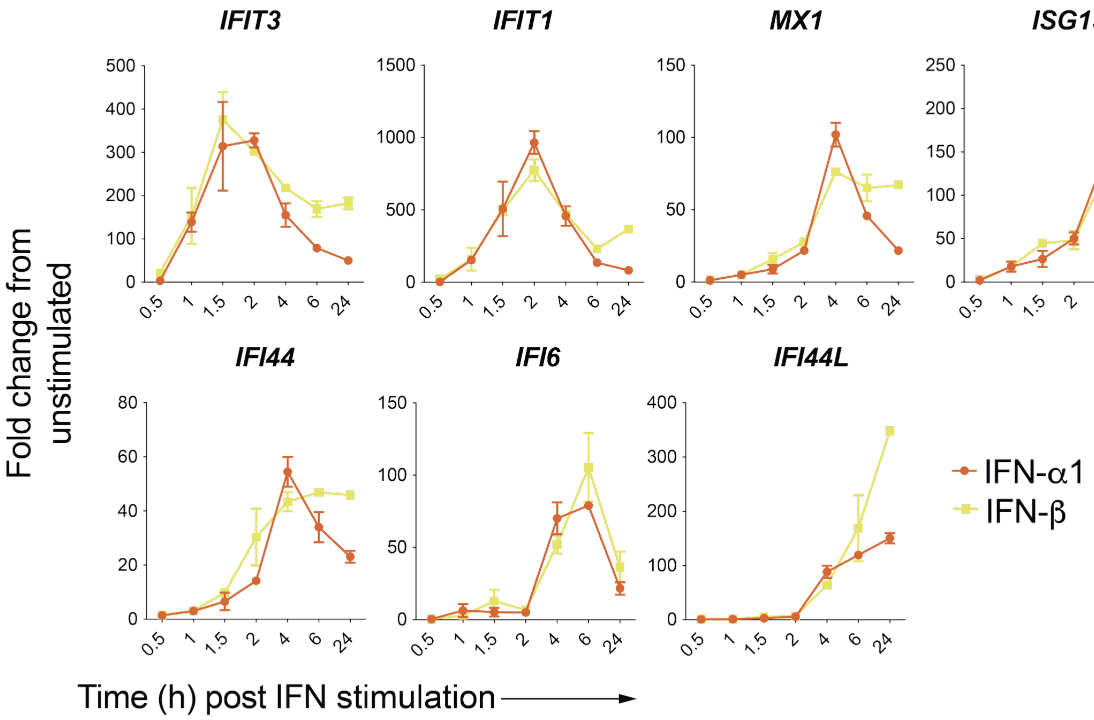

Appendix Figure S7 (Related to Figure 3). Kinetics of ISG expression in response to stimulation with IFN-α1 or IFN-β

PBMCs were stimulated with 250 U/ml of IFN-α1 or IFN-β or left unstimulated for the indicated periods of time. RNA was extracted and RT-qPCR for the indicated ISGs was performed. Data were normalised to expression of the housekeeping gene *HPRT* and are shown as fold change relative to unstimulated cells harvested at the same timepoint. Data are from PBMCs from one donor and error bars show range of duplicate stimulated wells.

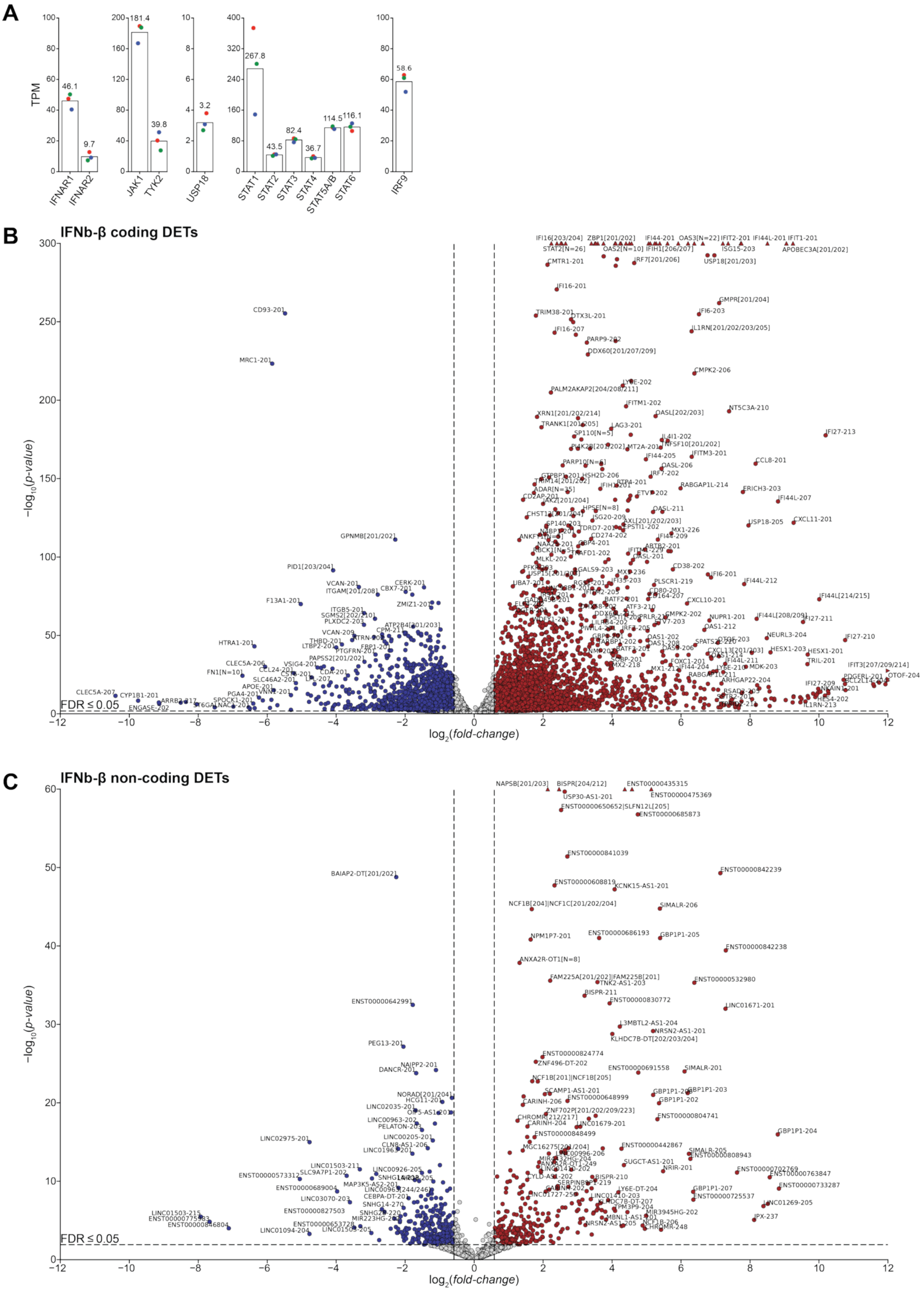

A
TPM
IFNAR1
IFNAR2
JAK1
TYK2
USP18
STAT1
STAT2
STAT3
STAT4
STAT5A/B
STAT6
IRF9
46.1
9.7
181.4
39.8
3.2
267.8
43.5
82.4
36.7
114.5
116.1
58.6
B
IFNb-β coding DETs
C
IFNb-β non-coding DETs
FDR ≤ 0.05
$-\log_{10}(p\text{-}value)$
$\log_2(fold\text{-}change)$

Appendix Figure S8 (Related to Figure 3). Differential expression of coding and non-coding RNAs in IFN-β-stimulated PBMCs

(A) Estimated expression levels (in TPM) of the indicated genes. Colours represent individual PBMC donors. (B-C) Volcano plots showing differentially expressed transcripts in IFN-β-stimulated PBMCs, corresponding to coding (B) and non-coding (C) genes. Triangles denote data points that fall outside the plot boundaries. Top transcripts are labelled with matched Ensembl names (when available) or Ensembl transcript IDs. For transcripts belonging to technically indistinguishable groups, all matched names are listed and separated by slashes (/); names sharing a common prefix are abbreviated using suffixes in brackets (e.g., RNA[10/20] for the RNA-10 and RNA-20 group). When more than five transcripts share the same prefix, the total number is indicated in brackets (e.g., RNA[N=10]).

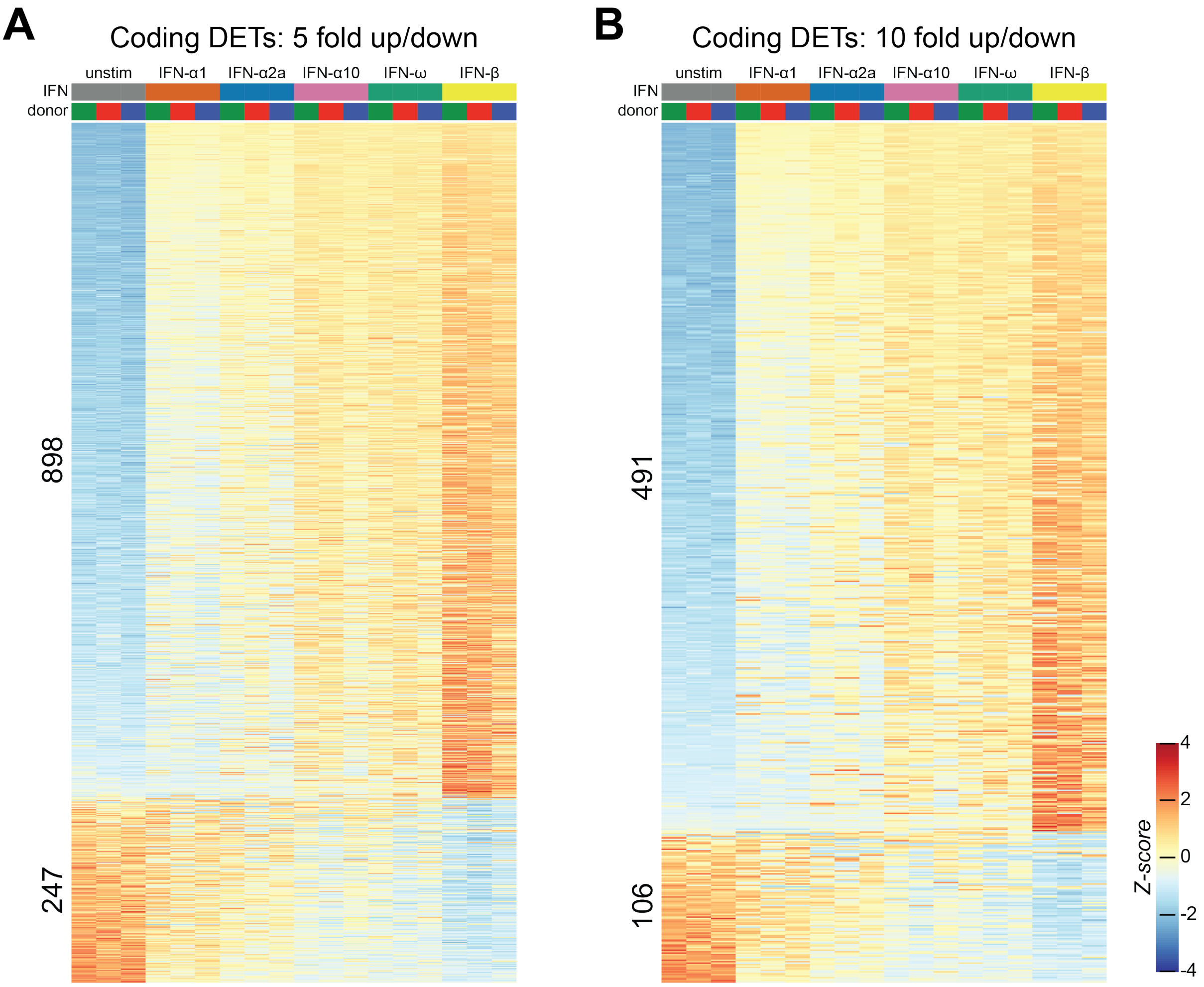

Appendix Figure S9 (Related to Figure 3). Number of differentially expressed coding RNAs using increased fold change stringency filters

(A,B) Heatmaps of transcripts mapping to coding genes that are differentially expressed in response to stimulation with at least one type I IFN (adj. p-value ≤ 0.05), using 5-fold (A) and 10-fold (B) thresholds. Transcripts are ranked by Z-score across all type I IFN-stimulated samples. The total number of up-regulated and down-regulated RNAs is indicated on the left side of each panel.

A

Up-regulated	Total number of transcripts	Number of unique transcripts
**IFN-α1**	1397	27
**IFN-α2a**	1790	23
**IFN-α10**	2724	60
**IFN-ω**	2880	80
**IFN-β**	4326	1331
**Transcripts up-regulated by all IFNs**		1240

Down-regulated	Total number of transcripts	Number of unique transcripts
**IFN-α1**	169	27
**IFN-α2a**	484	30
**IFN-α10**	1271	154
**IFN-ω**	1467	147
**IFN-β**	2950	1398
**Transcripts down-regulated by all IFNs**		103

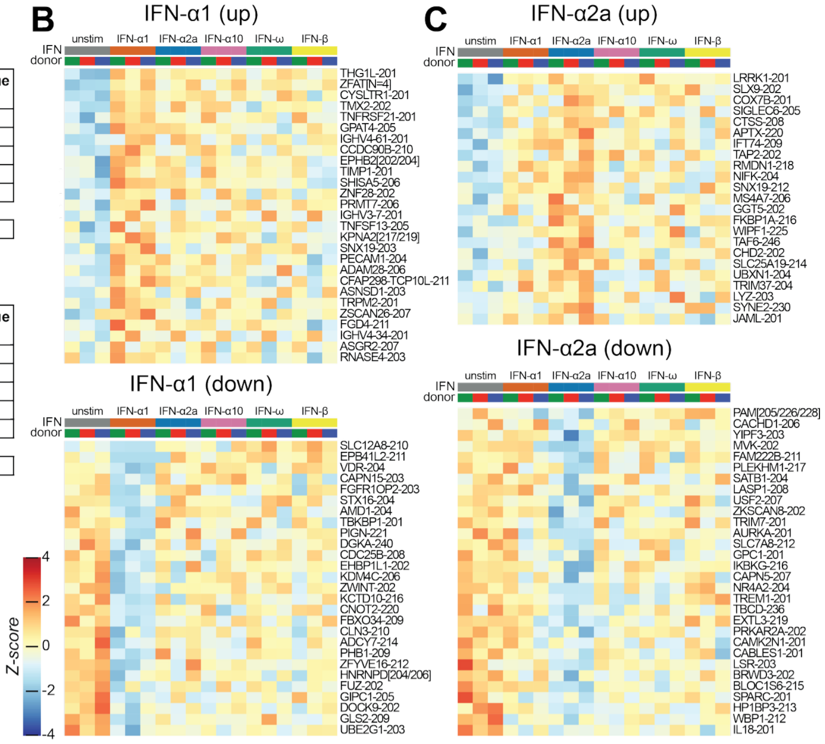

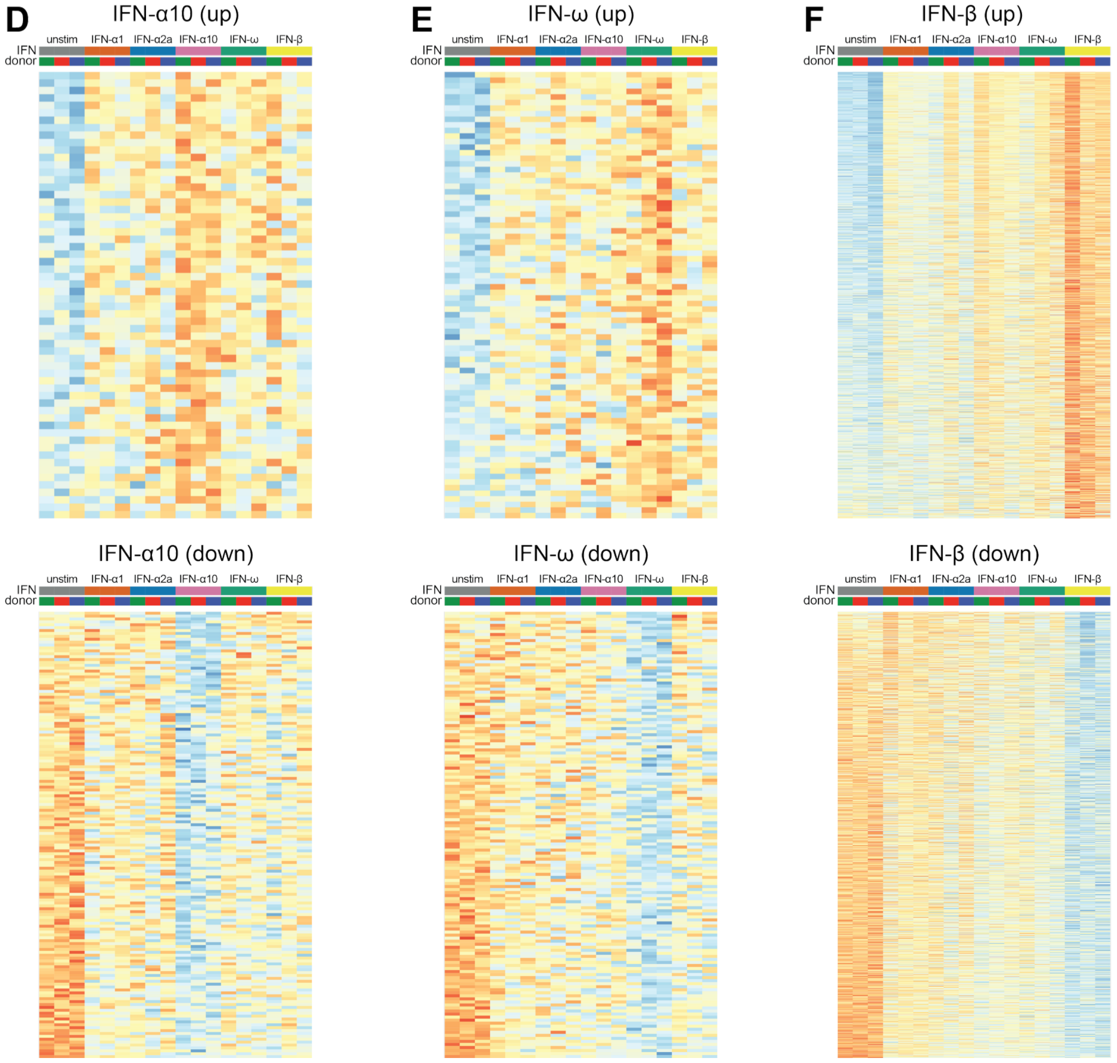

Appendix Figure S10 (Related to Figure 3). Coding RNAs differentially expressed in PBMCs following type I IFN treatment

(A) Total number of coding RNAs differentially expressed in PBMCs in response to each type I IFN subtype (adj. p-value <= 0.05, abs. fold-change >= 1.5). (B-F) Heatmaps of RNAs uniquely up- or down-regulated by the indicated type I IFN subtypes in PBMCs. In heatmaps with 50 rows or fewer, each transcript is labelled with a matched Ensembl name (when available) or its Ensembl transcript ID. For RNAs belonging to technically indistinguishable groups, all matched names are listed and separated by slashes; names sharing a common prefix are abbreviated using suffixes in brackets (e.g., RNA[10/20] for a RNA-10 and RNA-20 group). When more than five RNAs share the same prefix, the total number is indicated in brackets (e.g., RNA[N=10]). Colours indicate individual donors, and RNAs are ranked by Z-score across all type I IFN-stimulated samples.

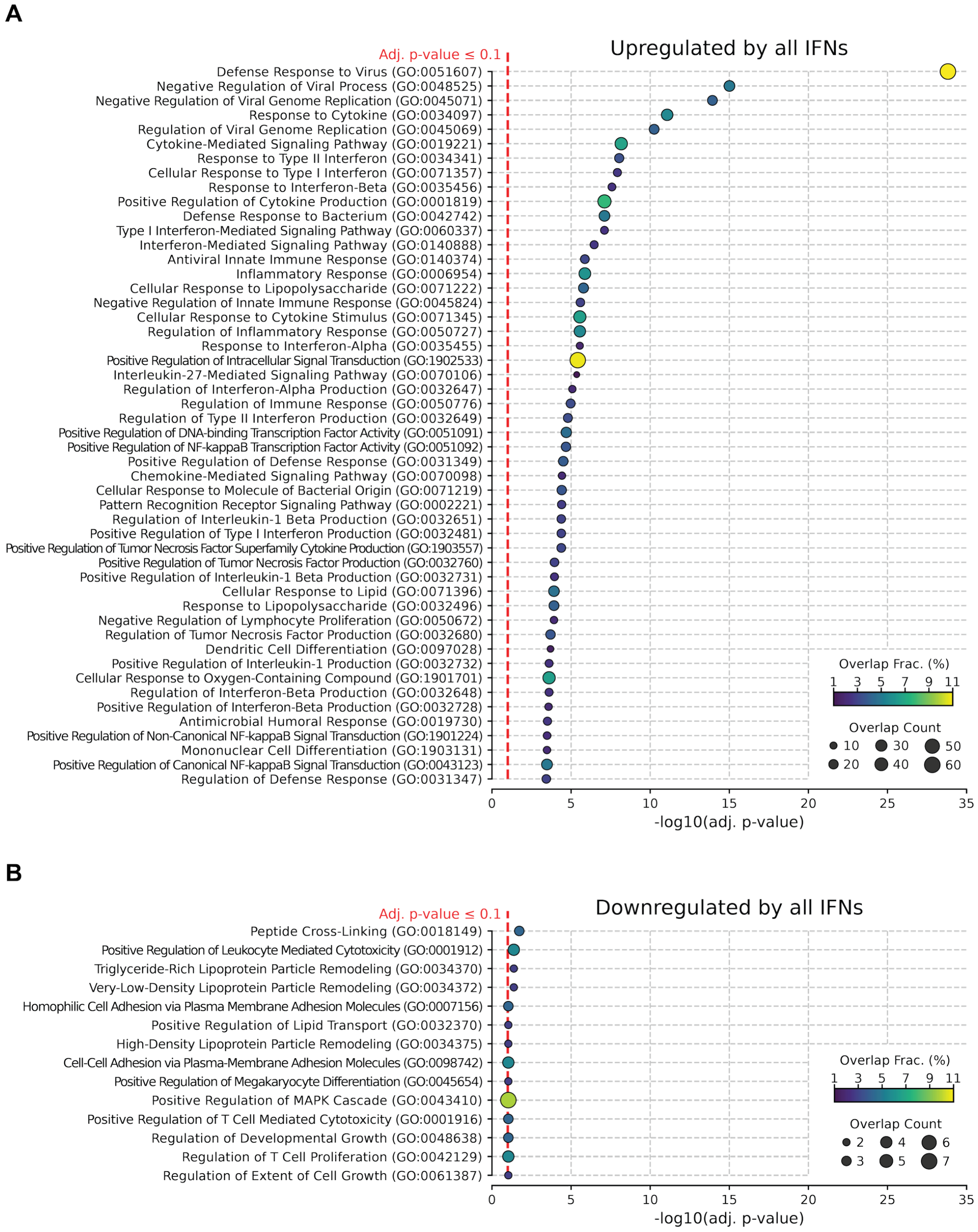
A
Upregulated by all IFNs
Adj. p-value ≤ 0.1
Defense Response to Virus (GO:0051607)
Negative Regulation of Viral Process (GO:0048525)
Negative Regulation of Viral Genome Replication (GO:0045071)
Response to Cytokine (GO:0034097)
Regulation of Viral Genome Replication (GO:0045069)
Cytokine-Mediated Signaling Pathway (GO:0019221)
Response to Type II Interferon (GO:0034341)
Cellular Response to Type I Interferon (GO:0071357)
Response to Interferon-Beta (GO:0035456)
Positive Regulation of Cytokine Production (GO:0001819)
Defense Response to Bacterium (GO:0042742)
Type I Interferon-Mediated Signaling Pathway (GO:0060337)
Interferon-Mediated Signaling Pathway (GO:0140888)
Antiviral Innate Immune Response (GO:0140374)
Inflammatory Response (GO:0006954)
Cellular Response to Lipopolysaccharide (GO:0071222)
Negative Regulation of Innate Immune Response (GO:0045824)
Cellular Response to Cytokine Stimulus (GO:0071345)
Regulation of Inflammatory Response (GO:0050727)
Response to Interferon-Alpha (GO:0035455)
Positive Regulation of Intracellular Signal Transduction (GO:1902533)
Interleukin-27-Mediated Signaling Pathway (GO:0070106)
Regulation of Interferon-Alpha Production (GO:0032647)
Regulation of Immune Response (GO:0050776)
Regulation of Type II Interferon Production (GO:0032649)
Positive Regulation of DNA-binding Transcription Factor Activity (GO:0051091)
Positive Regulation of NF-kappaB Transcription Factor Activity (GO:0051092)
Positive Regulation of Defense Response (GO:0031349)
Chemokine-Mediated Signaling Pathway (GO:0070098)
Cellular Response to Molecule of Bacterial Origin (GO:0071219)
Pattern Recognition Receptor Signaling Pathway (GO:0002221)
Regulation of Interleukin-1 Beta Production (GO:0032651)
Positive Regulation of Type I Interferon Production (GO:0032481)
Positive Regulation of Tumor Necrosis Factor Superfamily Cytokine Production (GO:1903557)
Positive Regulation of Tumor Necrosis Factor Production (GO:0032760)
Positive Regulation of Interleukin-1 Beta Production (GO:0032731)
Cellular Response to Lipid (GO:0071396)
Response to Lipopolysaccharide (GO:0032496)
Negative Regulation of Lymphocyte Proliferation (GO:0050672)
Regulation of Tumor Necrosis Factor Production (GO:0032680)
Dendritic Cell Differentiation (GO:0097028)
Positive Regulation of Interleukin-1 Production (GO:0032732)
Cellular Response to Oxygen-Containing Compound (GO:1901701)
Regulation of Interferon-Beta Production (GO:0032648)
Positive Regulation of Interferon-Beta Production (GO:0032728)
Antimicrobial Humoral Response (GO:0019730)
Positive Regulation of Non-Canonical NF-kappaB Signal Transduction (GO:1901224)
Mononuclear Cell Differentiation (GO:1903131)
Positive Regulation of Canonical NF-kappaB Signal Transduction (GO:0043123)
Regulation of Defense Response (GO:0031347)
Overlap Frac. (%)
1 3 5 7 9 11
Overlap Count
10 30 50
20 40 60
0 5 10 15 20 25 35
-log10(adj. p-value)
B
Downregulated by all IFNs
Adj. p-value ≤ 0.1
Peptide Cross-Linking (GO:0018149)
Positive Regulation of Leukocyte Mediated Cytotoxicity (GO:0001912)
Triglyceride-Rich Lipoprotein Particle Remodeling (GO:0034370)
Very-Low-Density Lipoprotein Particle Remodeling (GO:0034372)
Homophilic Cell Adhesion via Plasma Membrane Adhesion Molecules (GO:0007156)
Positive Regulation of Lipid Transport (GO:0032370)
High-Density Lipoprotein Particle Remodeling (GO:0034375)
Cell-Cell Adhesion via Plasma-Membrane Adhesion Molecules (GO:0098742)
Positive Regulation of Megakaryocyte Differentiation (GO:0045654)
Positive Regulation of MAPK Cascade (GO:0043410)
Positive Regulation of T Cell Mediated Cytotoxicity (GO:0001916)
Regulation of Developmental Growth (GO:0048638)
Regulation of T Cell Proliferation (GO:0042129)
Regulation of Extent of Cell Growth (GO:0061387)
Overlap Frac. (%)
1 3 5 7 9 11
Overlap Count
2 4 6
3 5 7
0 5 10 15 20 25 35
-log10(adj. p-value)

Appendix Figure S11 (Related to Figure 3). Gene Ontology (GO) enrichment of coding genes hosting RNAs differentially expressed in PBMCs across all type I IFN stimulations.

(A,B) Biological Process GO enrichment analysis of coding genes containing at least one transcript significantly upregulated (A) or downregulated (B) across all type I IFN treatments, relative to unstimulated PBMCs (adj. p-value ≤ 0.05; abs. fold change ≥ 1.5). Dot size represents the number of genes overlapping with each GO term (overlap count), and colour indicates the overlap fraction relative to the total number of up- or down-regulated genes. The top 50 significantly enriched GO terms are shown for upregulated genes (A), while all significant terms (adj. p-value ≤ 0.1) are shown for downregulated genes (B).

A

Up-regulated	Total number of transcripts	Number of unique transcripts
IFN-α1	89	0
IFN-α2a	124	1
IFN-α10	202	5
IFN-ω	212	9
IFN-β	318	102
Transcripts up-regulated by all IFNs		85

Down-regulated	Total number of transcripts	Number of unique transcripts
IFN-α1	18	0
IFN-α2a	46	4
IFN-α10	160	24
IFN-ω	162	20
IFN-β	325	147
Transcripts down-regulated by all IFNs		11

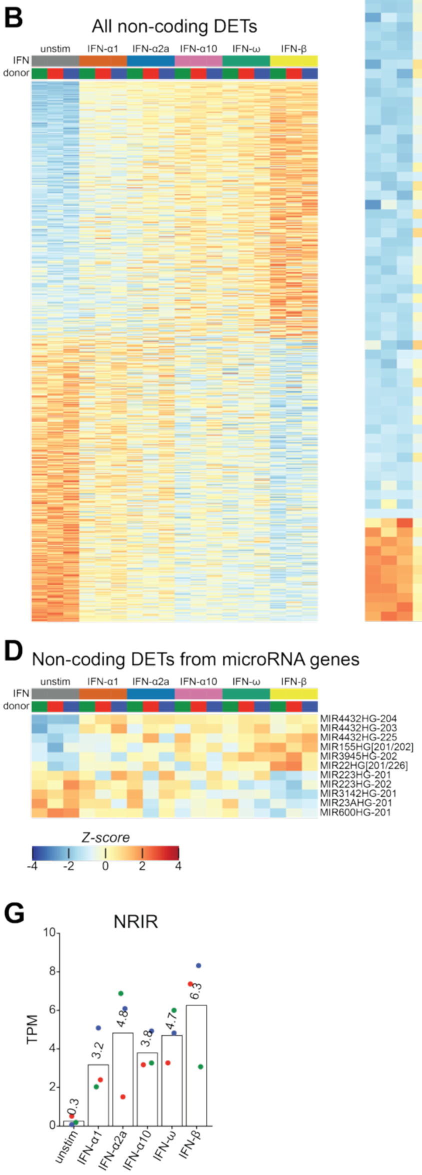

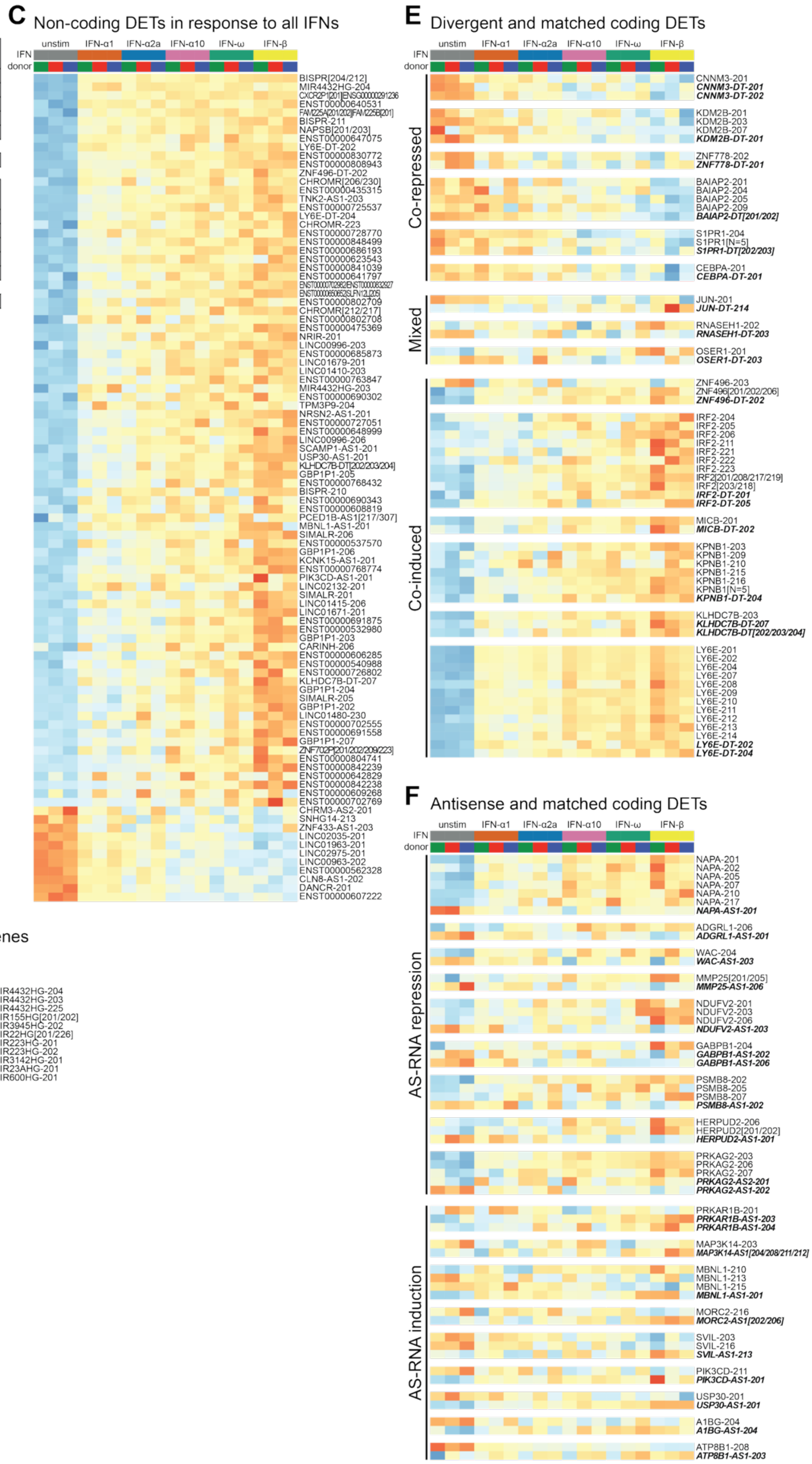

Appendix Figure S12 (Related to Figure 3). Non-coding RNAs differentially expressed in PBMCs following type I IFN treatment

(A) Total number of ncRNAs differentially expressed in PBMCs in response to each type I IFN subtype (adj. p-value <= 0.05, abs. fold-change >= 1.5). (B) Heatmap of all ncRNAs differentially expressed (adj. p-value ≤ 0.05; abs. fold change ≥ 1.5) in response to at least one type I IFN subtype, relative to unstimulated PBMCs (338 up-regulated and 379 down-regulated). (C) NcRNAs differentially expressed across all type I IFN subtypes. (D) NcRNAs mapping to microRNA genes differentially expressed in response to at least one type I IFN subtype. (E) Differentially expressed divergent and matched coding transcripts in response to at least one type I IFN subtype. Divergent transcripts are indicated in ***bold italics***. (F) Differentially expressed AS and matched coding transcripts in response to at least one type I IFN subtype. AS transcripts are indicated in ***bold italics***. (G) Estimated expression levels (in TPM) of the NRIR lncRNA. Colours represent individual PBMC donors.

In (B-F), colours indicate individual donors. In (B-D), RNAs are ranked by Z-score across all type I IFN-stimulated samples. In (E-F), RNAs are grouped by the parental gene and divergent (E) or AS (F) RNAs highlighted in bold. In (C-F), each transcript is labelled with a matched Ensembl name (when available) or its Ensembl transcript ID. For RNAs belonging to technically indistinguishable groups, all matched names are listed and separated by slashes; names sharing a common prefix are abbreviated using suffixes in brackets (e.g., RNA[10/20] for a RNA-10 and RNA-20 group). When more than five RNAs share the same prefix, the total number is indicated in brackets (e.g., RNA[N=10]).

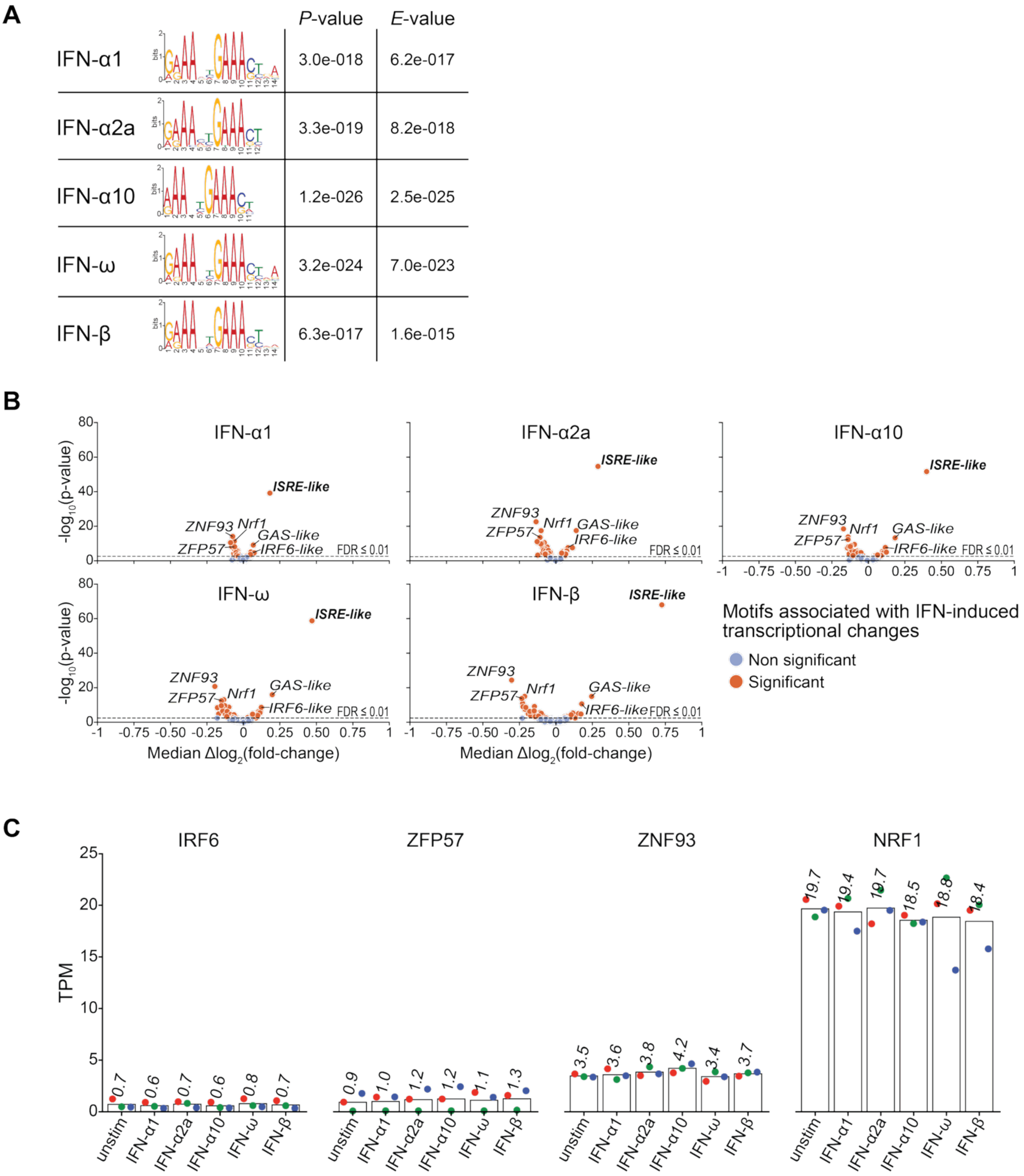

Appendix Figure S13 (Related to Figure 3). Promoter analysis of transcripts induced by type I IFN

(A) STREME motif analysis of promoter regions of transcripts significantly upregulated (adjusted p ≤ 0.05; fold change ≥ 1.5) following treatment with the indicated type I IFNs. The most enriched motif for each IFN treatment is shown alongside corresponding P- and E-values. (B) Association between JASPAR-annotated TF motif clusters and IFN-induced transcriptional changes in PBMCs. The horizontal axis shows the median Δ$\log_2$(fold-change) in RNA expression upon IFN treatment for transcripts whose promoters contain the respective TF motif (Z-score ≥ 2), compared to those lacking it (Z-score ≤ –1). Selected motifs consistently associated with transcriptional regulation across IFN types are labelled. (C) Estimated expression levels (in TPM) of the indicated genes. Colours represent individual PBMC donors.

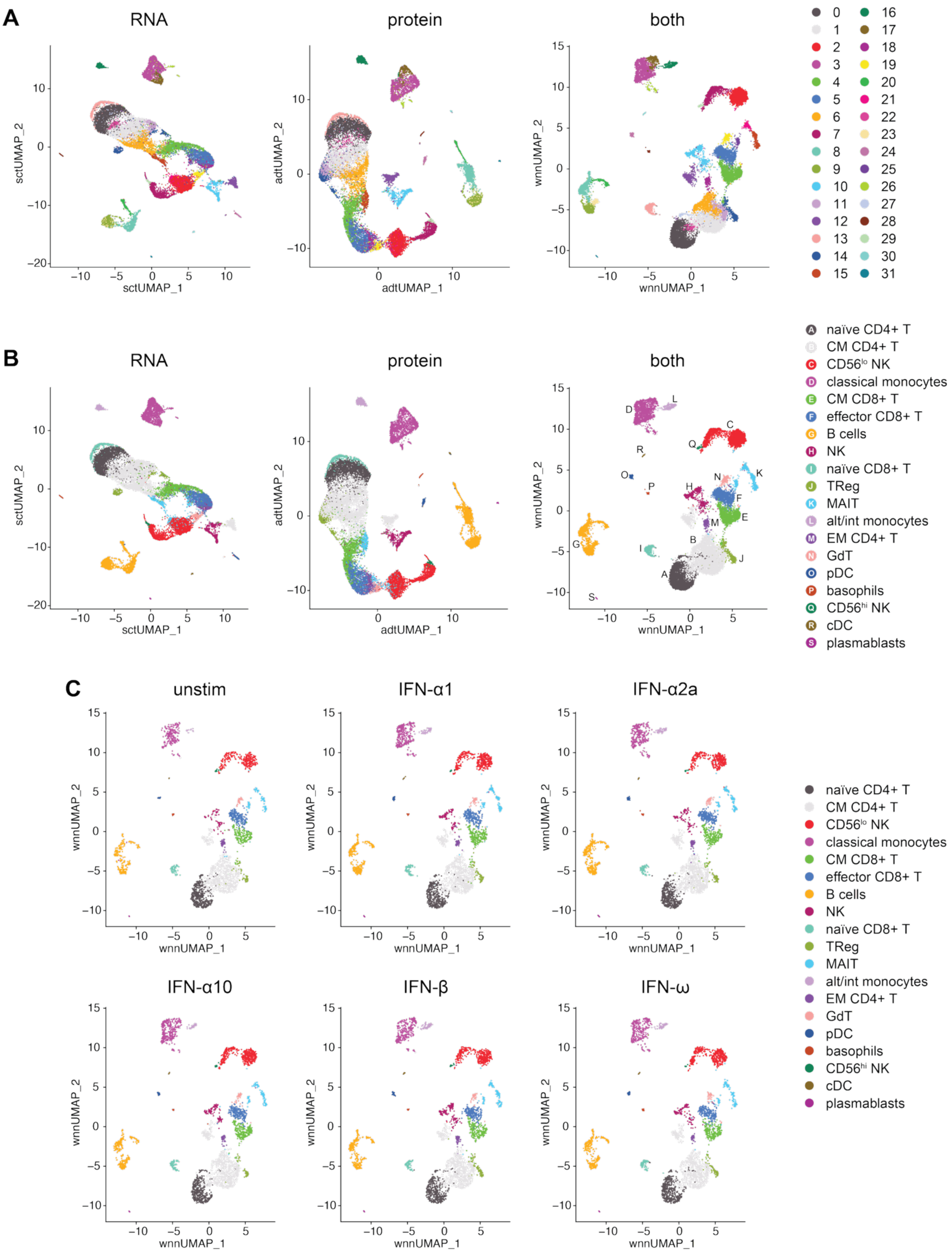

Appendix Figure S14 (Related to Figure 4). CITE-seq of PBMCs stimulated with type I IFNs
(A) UMAP plots showing the 32 cell clusters identified following clustering by RNA expression, protein expression or a weighted combination of both (cells from all samples combined). (B) Identification of different cell types based on merging of the clusters in (A). (C) Separation of the WNN UMAP by sample.

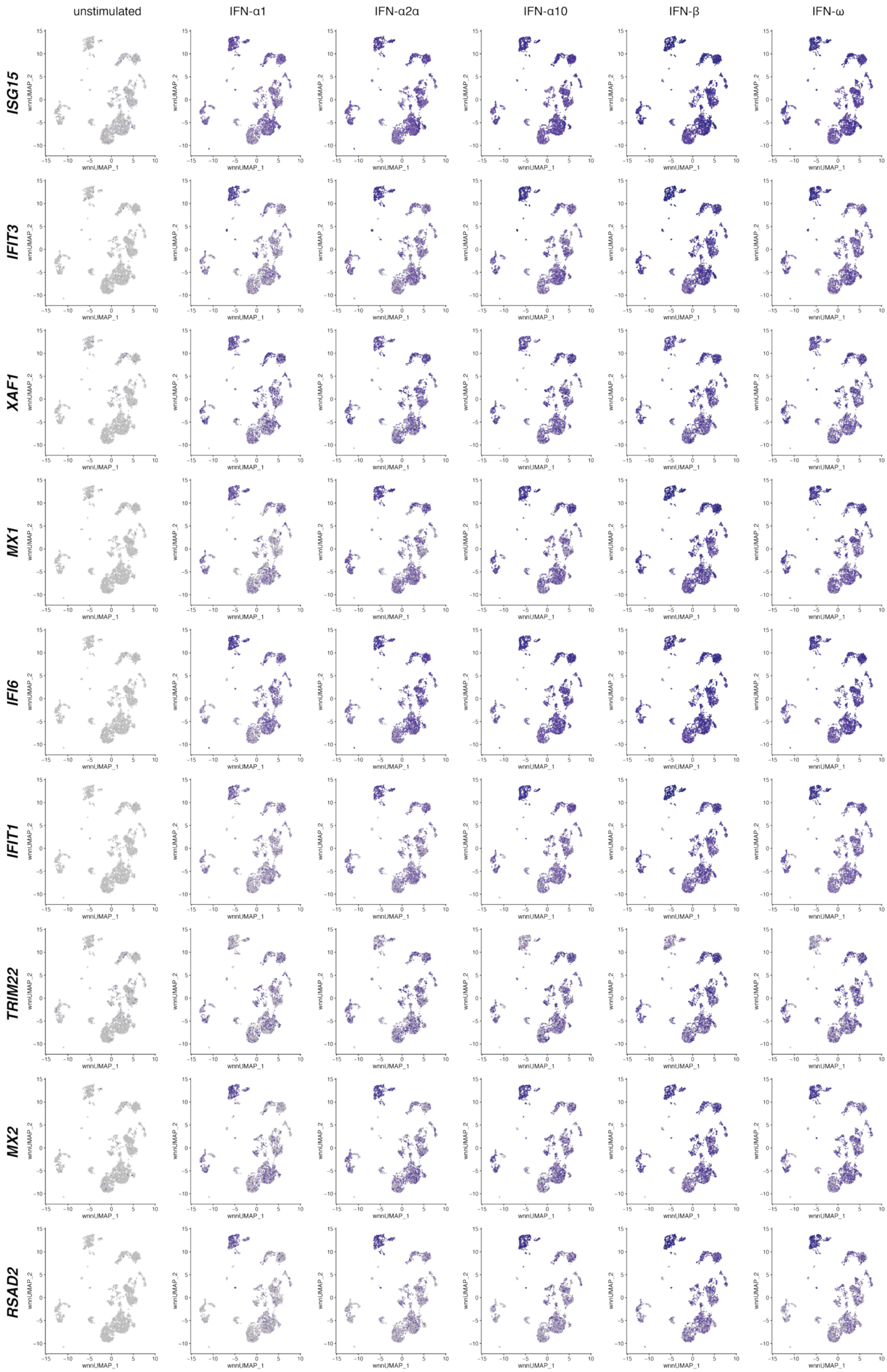
unstimulated
IFN-α1
IFN-α2α
IFN-α10
IFN-β
IFN-ω
ISG15
IFIT3
XAF1
MX1
IFI6
IFIT1
TRIM22
MX2
RSAD2
wnnUMAP_1
wnnUMAP_2

Appendix Figure S15 (Related to Figure 4). Core ISGs
UMAP plots showing the expression of the ten core ISGs across all samples.

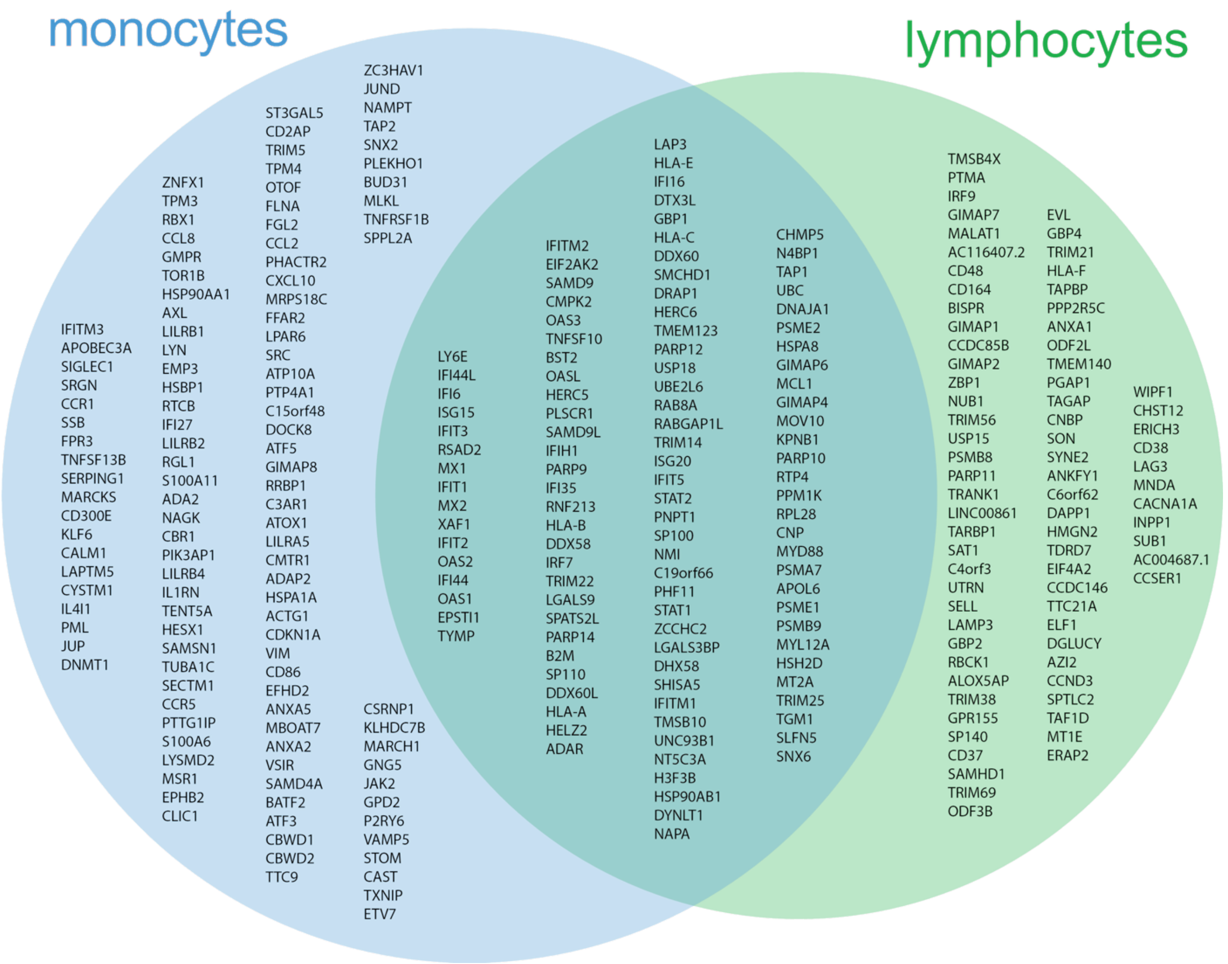

Appendix Figure S16 (Related to Figure 5). Monocyte and lymphocyte-specific ISGs
Venn diagram from Figure 5A showing the names of the genes in each segment.

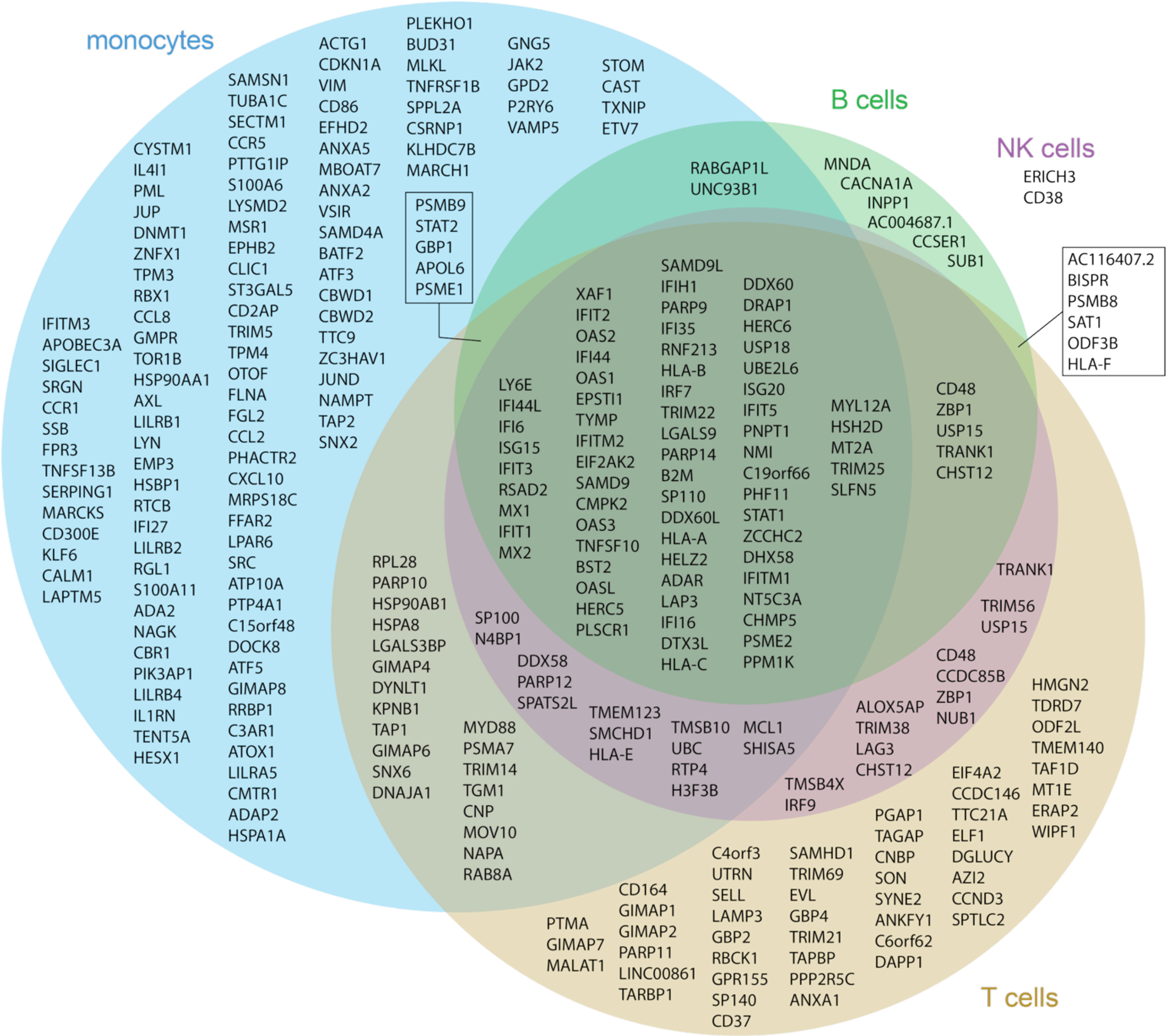

Appendix Figure S17 (Related to Figure 5). Monocyte and lymphocyte-specific ISGs

Venn diagrams showing cell type-specific ISGs as in Appendix Figure S16, further dividing lymphocytes into B cells, T cells and NK cells.

A

Monocytes	Total number of genes	Number of unique genes
**IFN-α1**	278	20
**IFN-α2a**	330	2
**IFN-α10**	482	9
**IFN-β**	643	143
**IFN-ω**	526	22

**Genes up-regulated by all IFNs**	225

Lymphocytes	Total number of genes	Number of unique genes
**IFN-α1**	185	1
**IFN-α2a**	299	0
**IFN-α10**	421	16
**IFN-β**	592	143
**IFN-ω**	475	38

**Genes up-regulated by all IFNs**	175

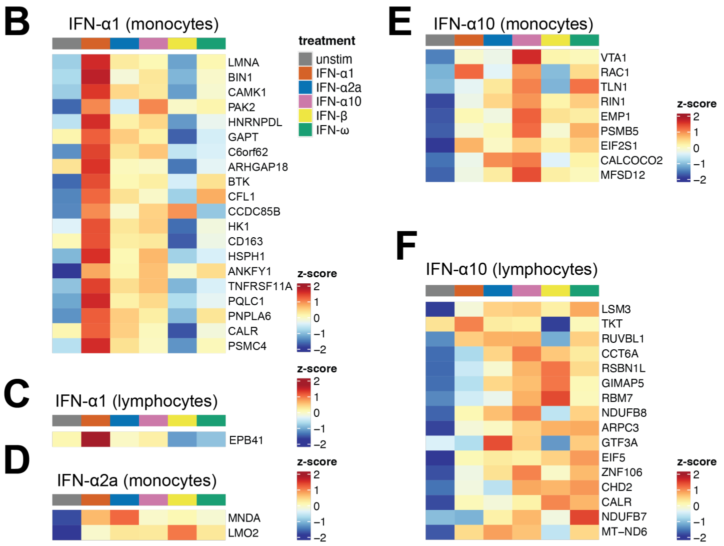

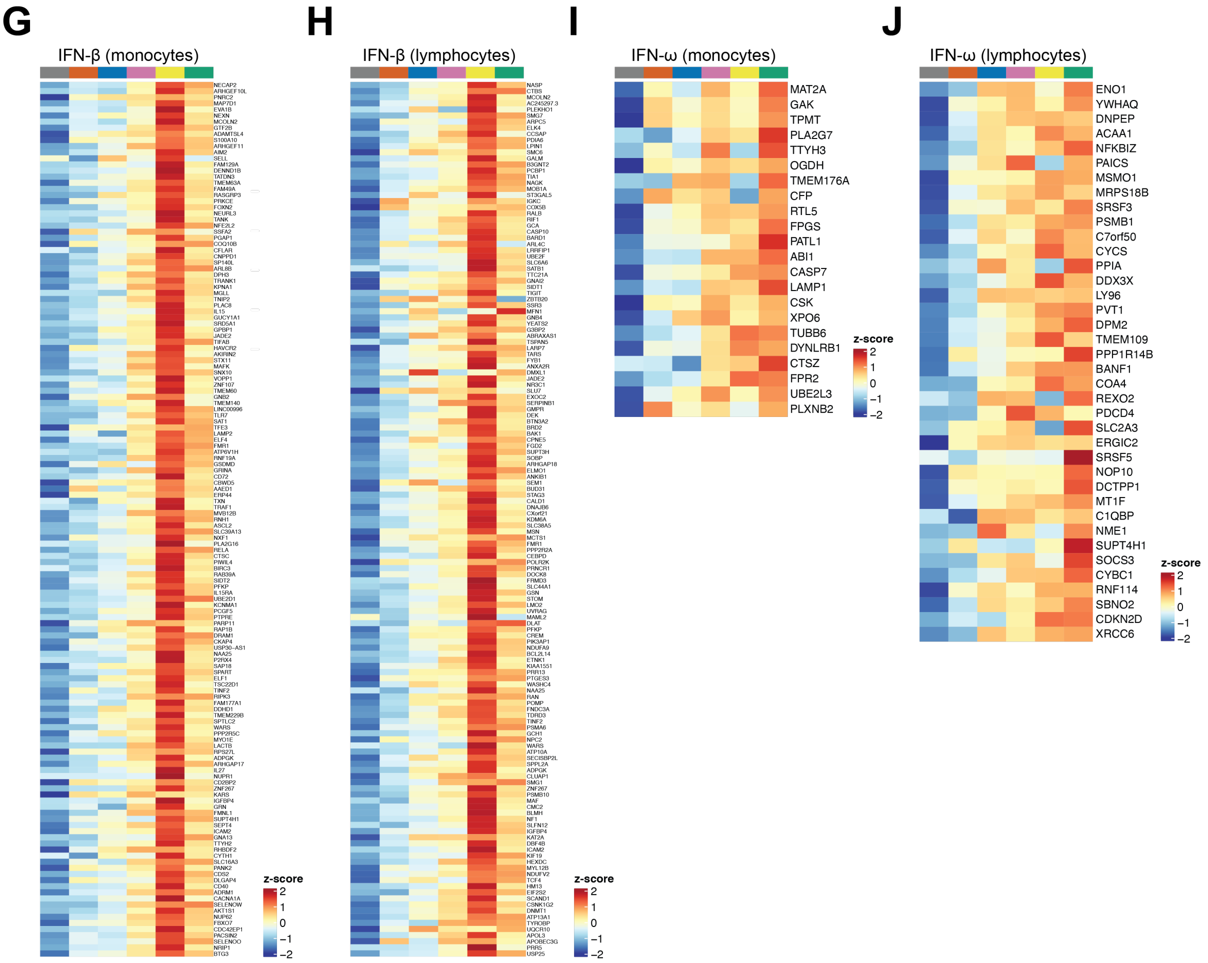

Appendix Figure S18 (Related to Figure 5). Genes up-regulated in response to only one type I IFN subtype only in monocytes or lymphocytes

(A) Summary tables of the number of differentially expressed genes in response to stimulation with each type I IFN subtype in monocytes (classical monocytes, alternative/intermediate monocytes) and lymphocytes (central memory CD4+ T cells, naïve CD4+ T cells, $CD56^{lo}$ NK cells, B cells, central memory CD8+ T cells, effector CD8+ T cells, MAIT cells, other NK cells, naïve CD8+ T cells, regulatory T cells, effector memory CD4+ T cells and γδ-T cells). (B-J) Heatmaps for genes uniquely up-regulated by the indicated type I IFN subtype compared to unstimulated PBMCs (padj < 0.05, $\log_2$ fold change > 0.25) plotted for myeloid cells (all monocytes, cDCs, basophils) or lymphocytes (all T cells, B cells, NK cells, pDCs). Genes were ranked by z-score for the indicated type I IFN-stimulated samples.

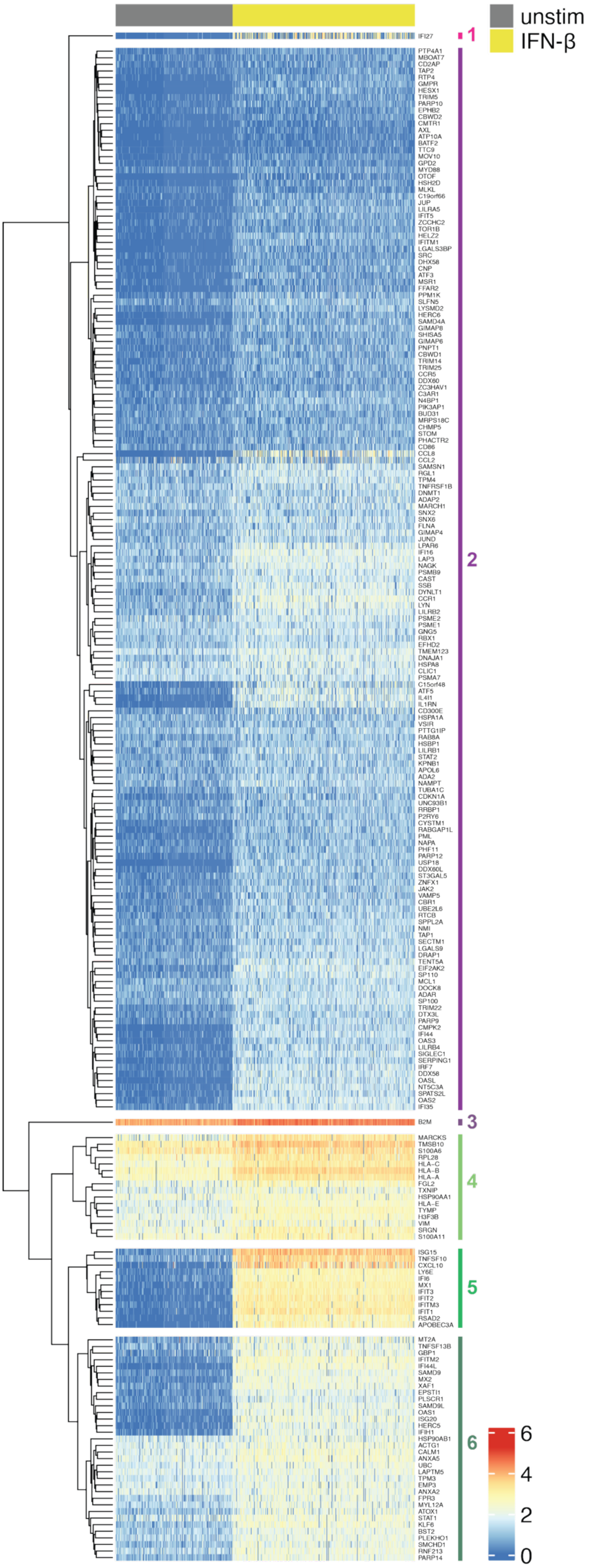
unstim
IFN-β
1
2
3
4
5
6
6
4
2
0
IFI27
PTP4A1
MBOAT7
CD2AP
TAP2
RTP4
GMPR
HESX1
TRIM5
PARP10
EPHB2
CBWD2
CMTR1
AXL
ATP10A
BATF2
TTC9
MOV10
GPD2
MYD88
OTOF
HSH2D
MLKL
C19orf66
JUP
LILRA5
IFIT5
ZCCHC2
TOR1B
HELZ2
IFITM1
LGALS3BP
SRC
DHX58
CNP
ATF3
MSR1
FFAR2
PPM1K
SLFN5
LYSMD2
HERC6
SAMD4A
GIMAP8
SHISA5
GIMAP6
PNPT1
CBWD1
TRIM14
TRIM25
CCR5
DDX60
ZC3HAV1
C3AR1
N4BP1
PIK3AP1
BUD31
MRPS18C
CHMP5
STOM
PHACTR2
CD86
CCL8
CCL2
SAMSN1
RGL1
TPM4
TNFRSF1B
DNMT1
ADAP2
MARCH1
SNX2
SNX6
FLNA
GIMAP4
JUND
LPAR6
IFI16
LAP3
NAGK
PSMB9
CAST
SSB
DYNLT1
CCR1
LYN
LILRB2
PSME2
PSME1
GNG5
RBX1
EFHD2
TMEM123
DNAJA1
HSPA8
CLIC1
PSMA7
C15orf48
ATF5
IL4I1
IL1RN
CD300E
HSPA1A
VSIR
PTTG1IP
RAB8A
HSBP1
LILRB1
STAT2
KPNB1
APOL6
ADA2
NAMPT
TUBA1C
CDKN1A
UNC93B1
RRBP1
P2RY6
CYSTM1
RABGAP1L
PML
NAPA
PHF11
PARP12
USP18
DDX60L
ST3GAL5
ZNFX1
JAK2
VAMP5
CBR1
UBE2L6
RTCB
SPPL2A
NMI
TAP1
SECTM1
LGALS9
DRAP1
TENT5A
EIF2AK2
SP110
MCL1
DOCK8
ADAR
SP100
TRIM22
DTX3L
PARP9
CMPK2
IFI44
OAS3
LILRB4
SIGLEC1
SERPING1
IRF7
DDX58
OASL
NT5C3A
SPATS2L
OAS2
IFI35
B2M
MARCKS
TMSB10
S100A6
RPL28
HLA-C
HLA-B
HLA-A
FGL2
TXNIP
HSP90AA1
HLA-E
TYMP
H3F3B
VIM
SRGN
S100A11
ISG15
TNFSF10
CXCL10
LY6E
IFI6
MX1
IFIT3
IFIT2
IFITM3
IFIT1
RSAD2
APOBEC3A
MT2A
TNFSF13B
GBP1
IFITM2
IFI44L
SAMD9
MX2
XAF1
EPSTI1
PLSCR1
SAMD9L
OAS1
ISG20
HERC5
IFIH1
HSP90AB1
ACTG1
CALM1
ANXA5
UBC
LAPTM5
TPM3
EMP3
ANXA2
FPR3
MYL12A
ATOX1
STAT1
KLF6
BST2
PLEKHO1
SMCHD1
RNF213
PARP14

Appendix Figure S19 (Related to Figure 6). Response of classical monocytes to type I IFNs
Enlargement of the heatmap in Figure 6A with annotated rows (for online viewing).

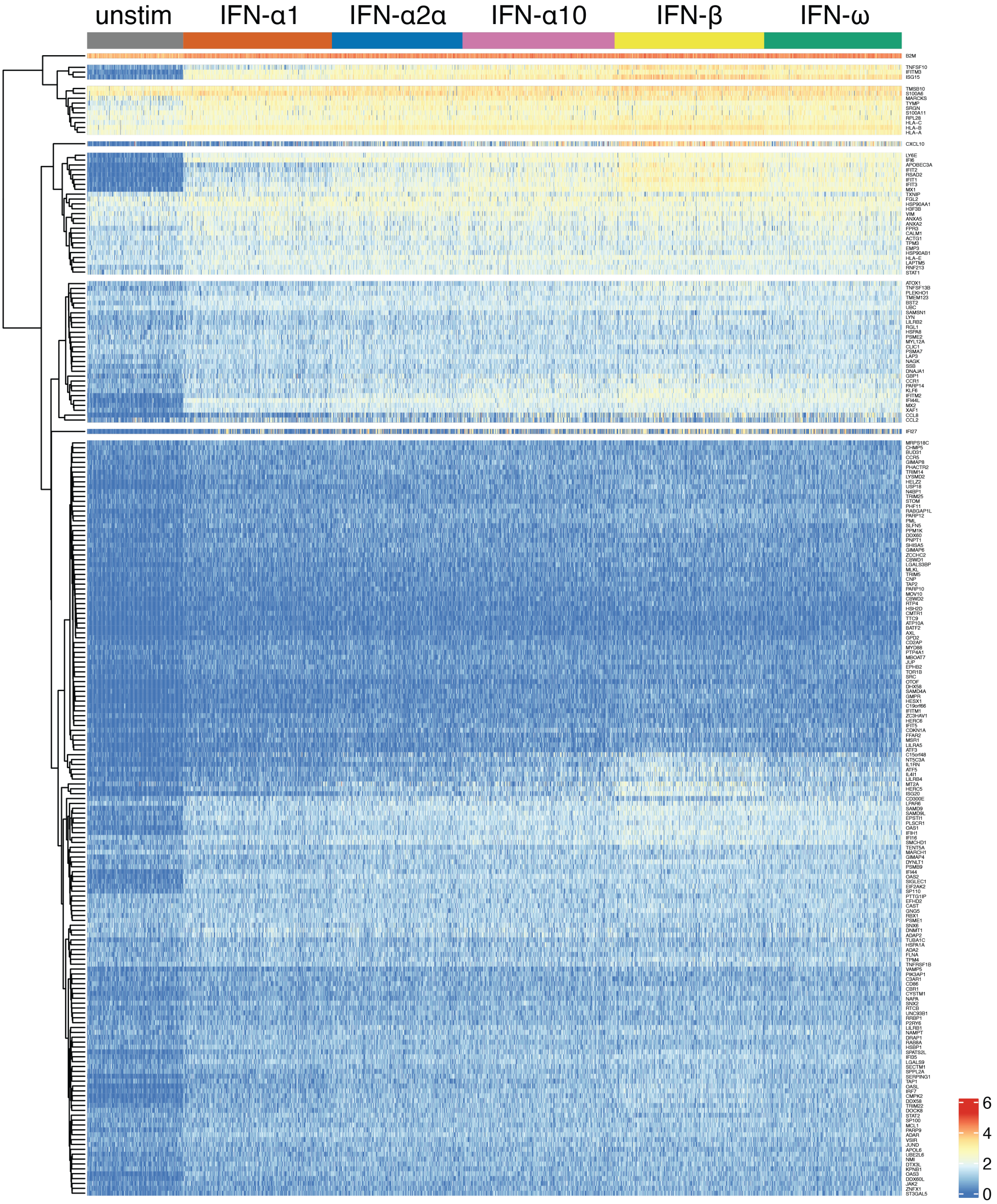

Appendix Figure S20 (Related to Figure 6). Response of classical monocytes to type I IFNs
Heatmap showing expression of the 225 genes significantly up-regulated in response to all tested type I IFNs in classical monocytes, in unstimulated and stimulated cells. Each row represents a gene and each column a cell.

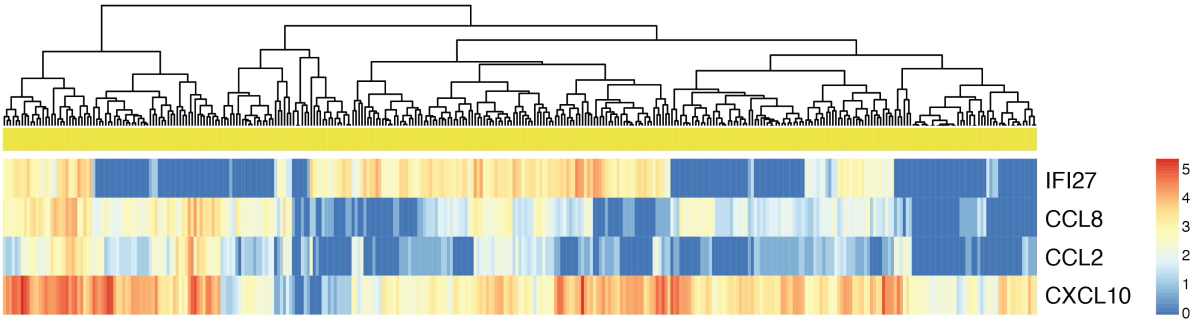

Appendix Figure S21 (Related to Figure 6). Expression of *IFI27*, *CCL8*, *CCL2* and *CXCL10* in individual cells

Expression of the indicated genes is shown for IFN-β-stimulated classical monocytes. Heatmaps are clustered by columns which represent individual cells.

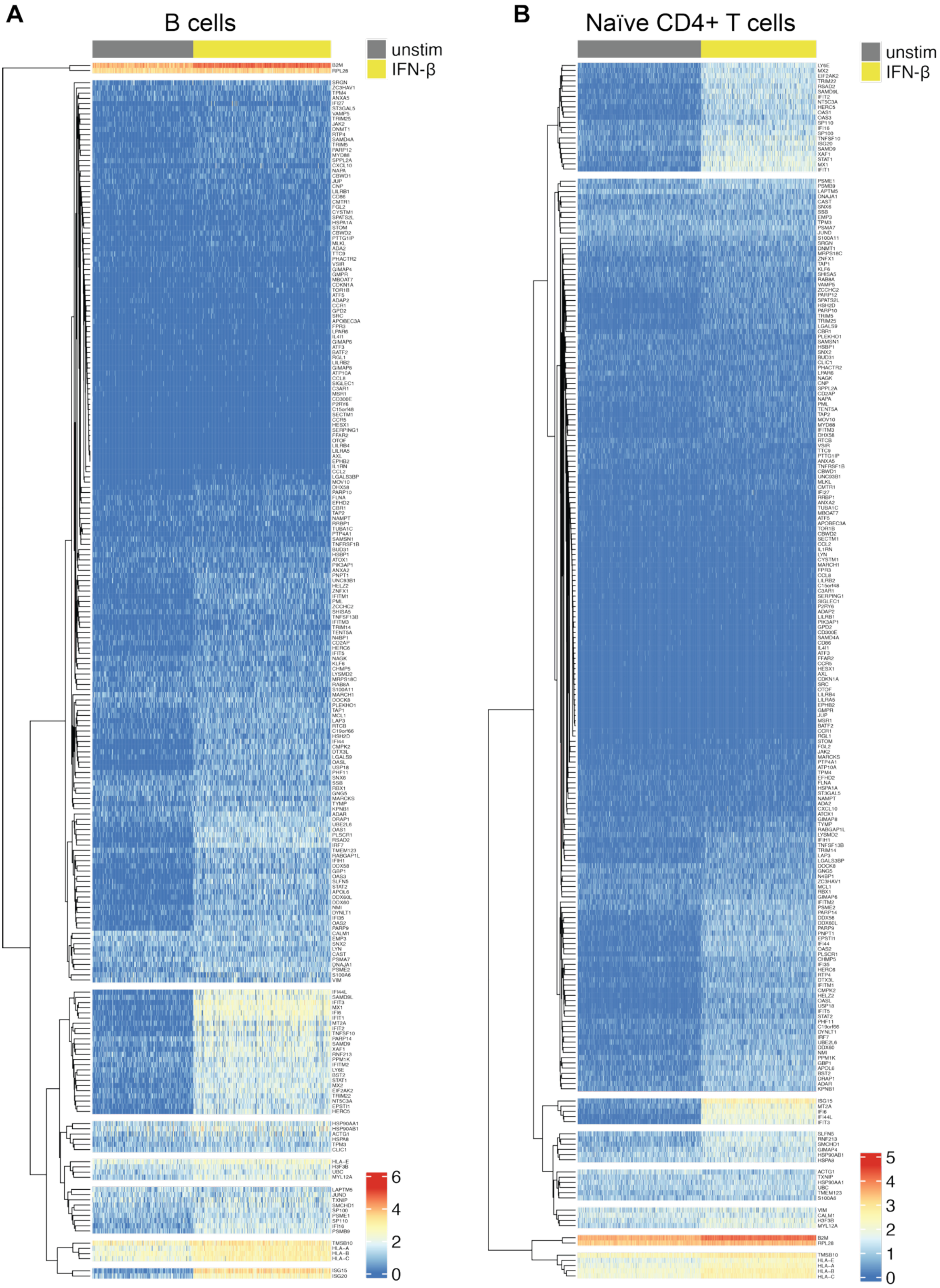

Appendix Figure S22 (Related to Figure 6). Expression of genes up-regulated in classical monocytes in other cell types

Heatmaps showing expression of the 225 genes that were significantly up-regulated by all tested type I IFNs in classical monocytes, in unstimulated and IFN-β-treated B cells and naïve CD4+ T cells. Gene names are provided for online viewing.

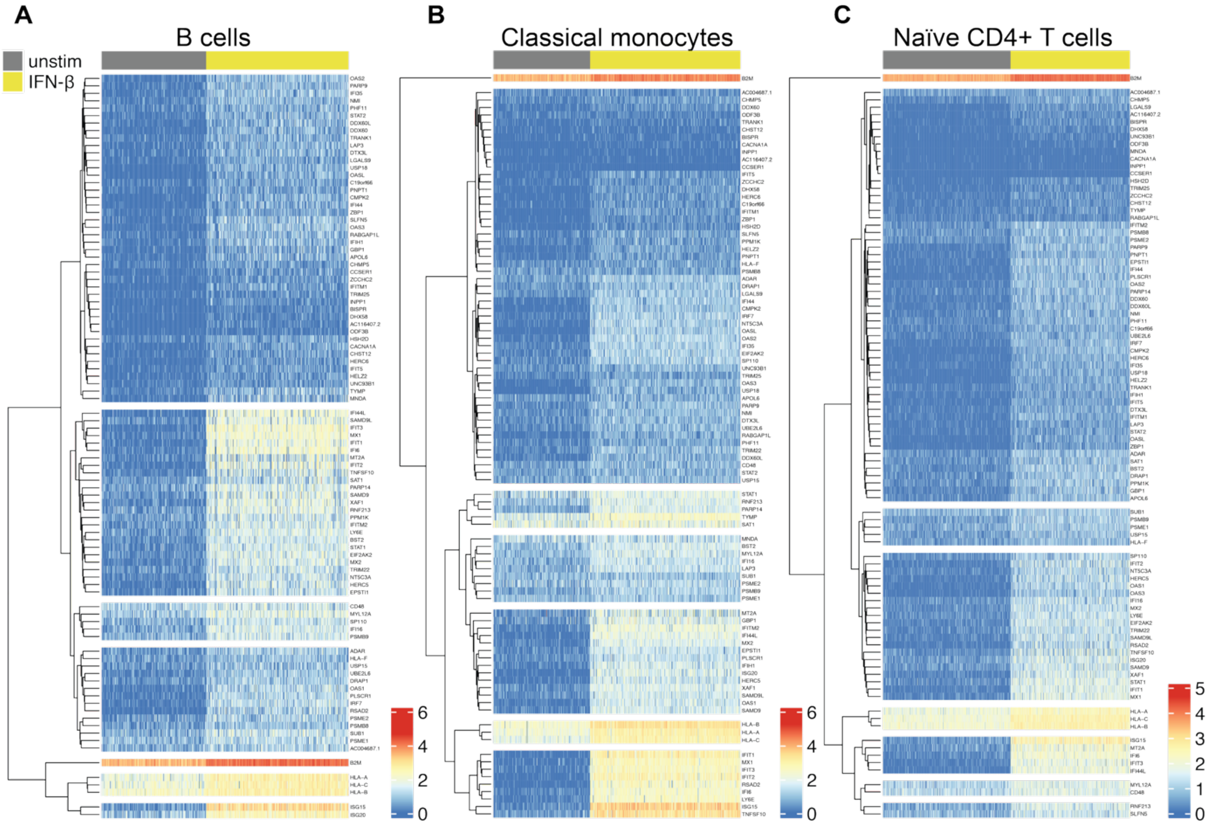

Appendix Figure S23 (Related to Figure 6). Response of B cells to type I IFNs

(A-C) Heatmaps showing expression of the 94 genes significantly up-regulated by all tested type I IFNs in B cells in unstimulated and IFN-β-treated cells for B cells (A), classical monocytes (B) and naïve CD4+ T cells (C). Gene names are provided for online viewing.

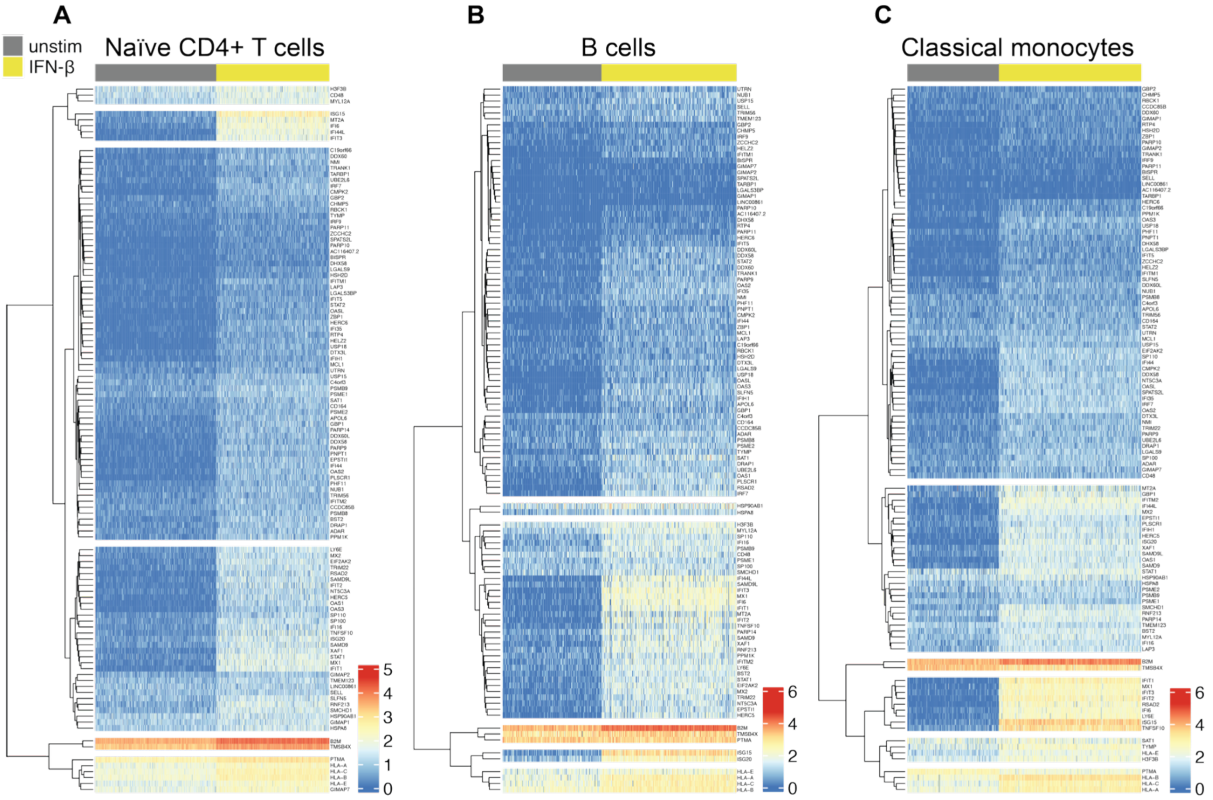

Appendix Figure S24 (Related to Figure 6). Response of naive CD4+ T cells to type I IFNs
(AC) Heatmaps showing expression of the 113 genes significantly up-regulated by all tested type I IFNs in naïve CD4+ T cells, in unstimulated and IFN-β-treated cells for naïve CD4+ T cells (A), B cells (B) and classical monocytes (C). Gene names are provided for online viewing.

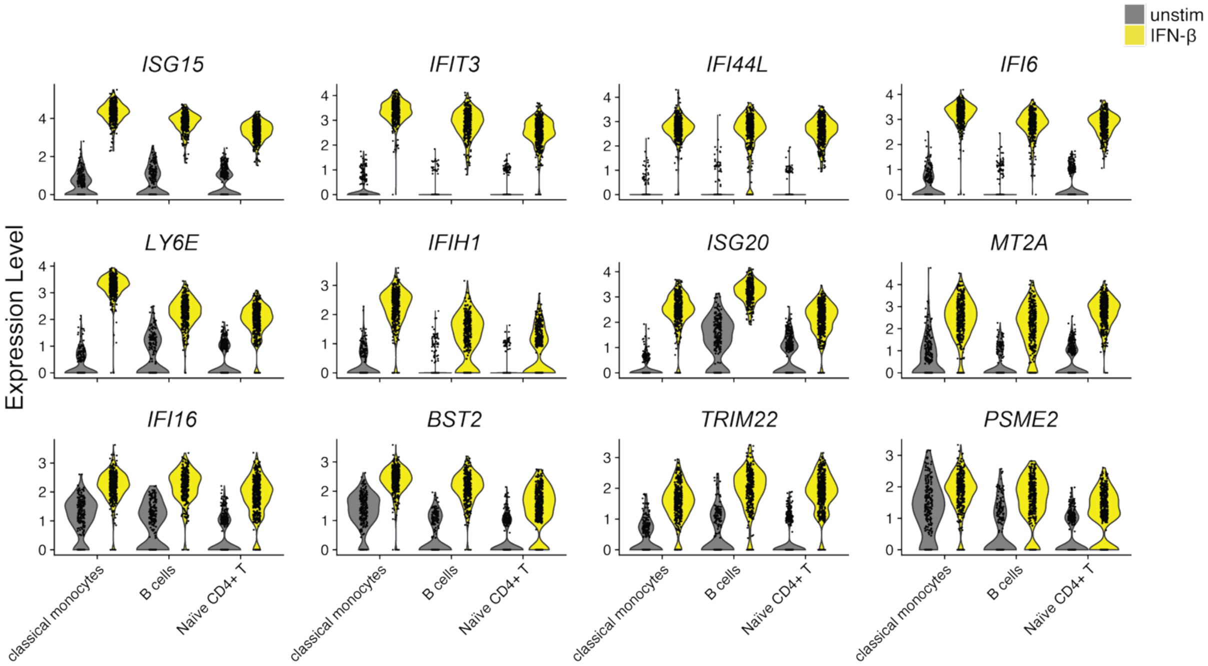

Appendix Figure S25 (Related to Figure 6). Selected ISGs significantly up-regulated by classical monocytes, B cells and naïve CD4+ T cells

Violin plots showing expression of twelve ISGs significantly up-regulated by classical monocytes, B cells and naïve CD4+ T cells in response to all type I IFN subtypes tested, in unstimulated and IFN-β-stimulated cells.

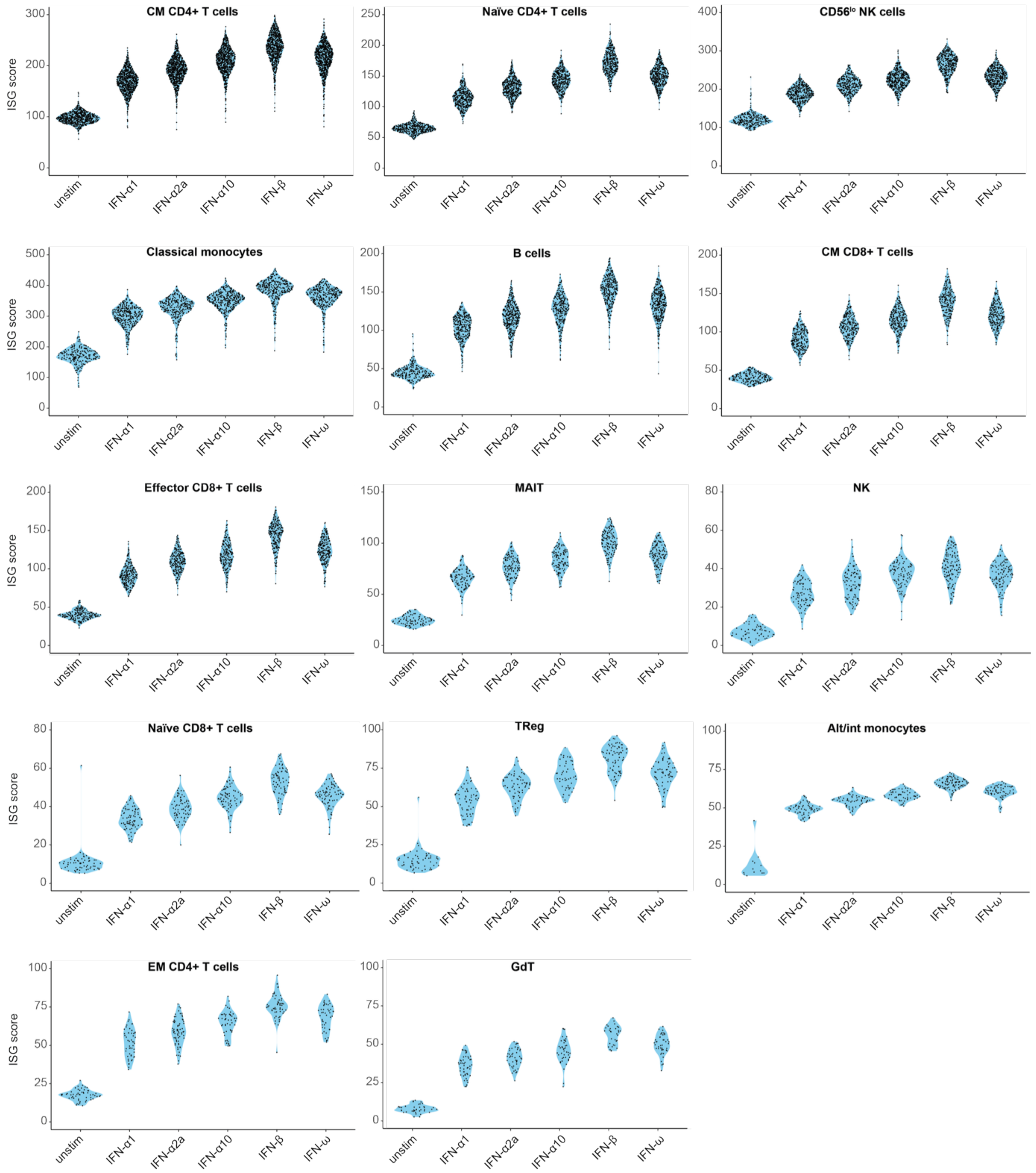

Appendix Figure S26 (Related to Figure 6). ISG scores for each cell type

Sinaplots with violin outline showing the ISG scores calculated using all genes significantly up-regulated by all type I IFNs for each cell type.

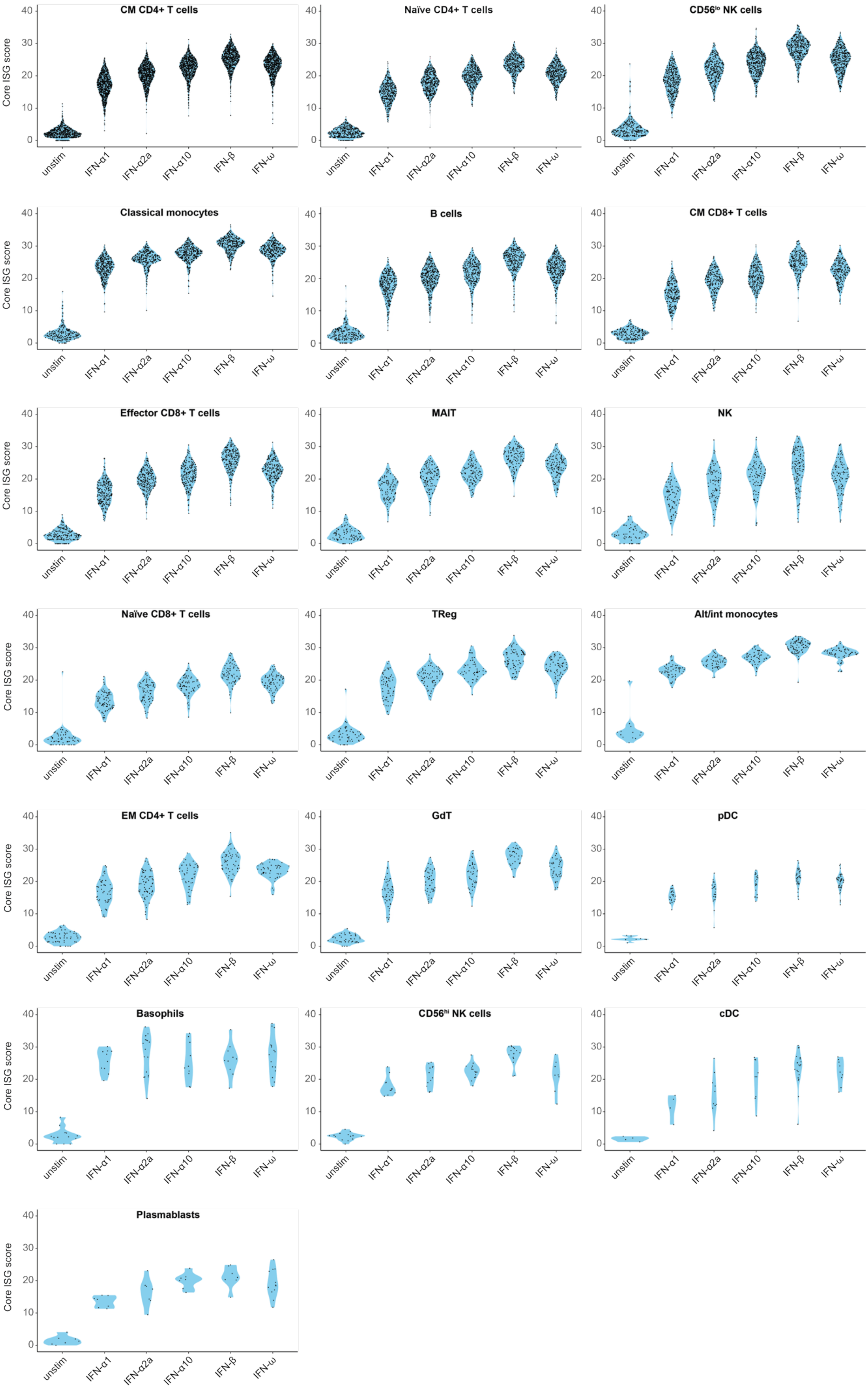
CM CD4+ T cells
Naïve CD4+ T cells
CD56lo NK cells
Classical monocytes
B cells
CM CD8+ T cells
Effector CD8+ T cells
MAIT
NK
Naïve CD8+ T cells
TReg
Alt/int monocytes
EM CD4+ T cells
GdT
pDC
Basophils
CD56hi NK cells
cDC
Plasmablasts
Core ISG score
0
10
20
30
40
unstim
IFN-α1
IFN-α2a
IFN-α10
IFN-β
IFN-ω

Appendix Figure S27 (Related to Figure 6). Core ISG scores for each cell type
Sinaplots with violin outline showing the ISG scores calculated using our ten core ISGs across each cell type.
